# PARSEC.py: A Python‐Based Real‐Space Kohn–Sham Density Functional Theory Code Accelerated by Machine Learned Charge Density

**DOI:** 10.1002/jcc.70482

**Published:** 2026-08-21

**Authors:** Zeyi Zhang, Carlos Mora Perez, Patrick Kwon, Martin Head‐Gordon, Jin Qian

**Affiliations:** ^1^ Chemical Sciences Division Lawrence Berkeley National Laboratory Berkeley California USA; ^2^ Pitzer Center for Theoretical Chemistry, Department of Chemistry University of California, Berkeley California USA; ^3^ College of Computing, Data Science and Society University of California, Berkeley California USA

**Keywords:** electronic structure theory, high performance computing, machine learning charge density, real‐space KS‐DFT

## Abstract

PARSEC.py is a Python‐based real‐space Kohn–Sham density functional theory (real‐space KS‐DFT) framework designed to provide a user‐ and developer‐friendly platform for first‐principles electronic‐structure simulations. Discretization on real‐space grids eliminates basis‐set approximations, while enabling systematic control of numerical accuracy and large‐scale parallelization. Building upon the theoretical foundations of the original PARSEC code, the framework leverages the Python scientific ecosystem to support modular development and integration with modern machine learning (ML) tools. PARSEC.py incorporates ML‐predicted density as an alternative initial guess for the superposition of atomic density (SAD) in self‐consistent field (SCF) iterations. This capability provides a framework for integrating ML models into electronic‐structure calculations and offers a pathway for accelerating SCF convergence. We present the theoretical framework, key algorithms, and performance characteristics of PARSEC.py using both SAD and ML initializations.

## Graphical abstract

PARSEC.py is a Python‐based real‐space Kohn–Sham DFT framework that leverages the Python scientific ecosystem for transparent, modular integration of machine‐learned densities and GPU acceleration. This unified design enables more efficient and scalable first‐principles simulations of large chemical and materials systems.

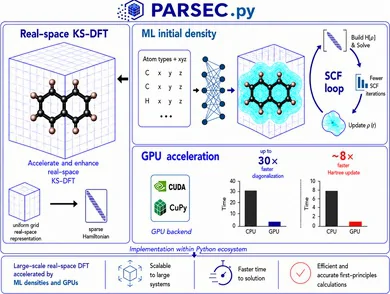

**Abbreviations**
AE: all electron
DFT: density functional theory
FD: finite difference
KS: Kohn–Sham
ML: machine learning
PP: pseudopotential
SAD: superposition of atomic density
SCF: self‐consistent field

## 1 Introduction

Kohn–Sham density functional theory (KS‐DFT) [1, 2] is a cornerstone of modern electronic‐structure modeling, offering a practical balance between accuracy and computational efficiency for a wide range of molecular and condensed‐phase systems [3]. Conventional DFT implementations, which employ basis functions such as plane waves in VASP [4, 5] and Quantum Espresso [6] or Gaussian‐type orbitals (GTOs) in Qchem [7] and Gaussian [8], have been instrumental in advancing quantum chemistry and materials science. However, their global‐basis representations and dense Hamiltonian matrices often limit scalability, particularly for systems with thousands of atoms or more complex interfacial environments.

Real‐space formulations of DFT provide an attractive alternative [9]. By discretizing the KS equations directly on finite‐difference (FD) [10, 11] or finite‐element (FE) [12, 13] grids, these methods yield sparse Hamiltonian matrices that are ideally suited for large‐scale parallel computation on modern high‐performance computing architectures [14]. Over the past two decades, several high‐performance real‐space KS‐DFT programs have demonstrated the power of this approach. The PARSEC code pioneered large‐scale FD simulations [15]; OCTOPUS extended the framework to time‐dependent DFT [16, 17]; SPARC [18, 19], ARES [20], and RESCU [21] achieved efficient scaling for systems containing tens of thousands of atoms. DFT‐FE introduced a FE formulation capable of all‐electron calculations [22, 23]. Despite these achievements, most existing real‐space codes are implemented in languages such as Fortran or C++, which, while highly efficient and performance‐oriented, often involve complex syntax, manual memory management, and intricate build systems. These characteristics make implementation, code modification, and method development more challenging, particularly for non‐specialists or researchers without extensive experience in low‐level programming.

In recent years, the Python programming language has emerged as a powerful tool for scientific computing, combining simplicity, readability, and flexibility with access to high‐performance libraries. Python's extensive ecosystem, including packages such as NumPy [24], SciPy [25], MPI4py [26], CuPy [27], and PyTorch [28], provides both ease of prototyping and seamless integration with optimized native backends. In electronic‐structure theory, Python has been widely used as a scripting language for workflow management in packages such as Psi4 [29], ASE [30], and pymatgen [31]. It has also been employed for symbolic implementations, for example, in the Tensor Contraction Engine [32] and SecondQuantizationAlgebra library [33]. Python has also begun to be used for implementing core electronic‐structure algorithms. Projects such as PySCF [34, 35] and GPAW [36, 37] have demonstrated that frameworks written primarily in Python can achieve computational performance comparable to traditional Fortran or C++ codes while improving accessibility and extensibility.

Moreover, the rapid emergence of AI for science (AI4Science) [38, 39, 40, 41, 42, 43], particularly ML approaches for electronic‐structure theory [44, 45, 46, 47, 48], has led to increasingly fast models capable of predicting fundamental quantities such as potential energy surfaces [49, 50, 51, 52], wavefunctions [53, 54, 55, 56], Hamiltonians [57, 58, 59, 60], and electron densities [61, 62, 63, 64]. These models can provide physically informed initial guesses that are often close to the final converged solutions, thereby significantly accelerating electronic‐structure calculations. As a result, the integration of ML frameworks into quantum chemistry workflows has attracted growing interest, enabling enhanced computational efficiency without sacrificing accuracy. Python has become the dominant development language for modern AI and ML frameworks. Consequently, quantum chemistry programs implemented in Python offer a natural and flexible platform for incorporating these methods. Such integration enables the development of new computational workflows that combine the predictive power of AI/ML models with the robustness and accuracy of traditional quantum‐chemical methods.

Although, ML models can in principle be coupled to existing Fortran or C++ electronic‐structure codes through file‐based interfaces, such loose coupling becomes cumbersome when ML predictions are used as active components of the electronic‐structure workflow rather than as simple pre‐ or post‐processing tools. For real‐space ML‐density initialization, the predicted density must be mapped onto the same grid, normalized, converted into Hartree and exchange‐correlation potentials, and incorporated into the SCF loop. Repeated transfer of large three‐dimensional arrays between an external DFT executable and a Python ML framework can introduce substantial I/O overhead, grid‐format conversion, CPU–GPU data movement, and bookkeeping complexity. A Python‐native framework avoids many of these barriers by keeping the density, potentials, wavefunctions, diagonalization routines, and ML models in a common numerical environment based on NumPy, SciPy, CuPy, and PyTorch‐compatible data structures. Therefore, PARSEC.py is intended not as a replacement for highly optimized production real‐space codes, but as a transparent and extensible platform for developing and benchmarking ML‐assisted real‐space KS‐DFT algorithms.

Beyond ML‐assisted initialization, another route to improving the efficiency of real‐space KS‐DFT is to reduce the cost of each SCF step through GPU acceleration. For example, CuPy [27] provides a path for porting selected computational kernels to NVIDIA GPUs while maintaining a unified high‐level code structure. This is particularly beneficial for real‐space methods, in which the dominant workloads often arise from repeated Hamiltonian operations and Hartree‐potential updates across many grids. At the same time, ongoing advances in GPU hardware and exascale‐oriented computing architectures suggest considerable future potential for large‐scale parallelization [65, 66, 67, 68].

Motivated by these developments, we have developed PARSEC.py, a real‐space KS‐DFT framework implemented primarily in Python. Building upon the theoretical foundations of the original PARSEC code, PARSEC.py aims to provide a modern, open‐source environment for first‐principles simulations directly on real‐space grids. The framework, including high‐level control, data handling, core algorithms, and workflow logic, is implemented in Python or using Python packages, ensuring clarity, flexibility, and ease of extension. This design achieves both transparency and performance, enabling efficient simulations while remaining highly accessible to new users and developers. Beyond supporting conventional real‐space KS‐DFT workflows, PARSEC.py is designed to incorporate ML‐predicted densities to assist electronic‐structure calculations, providing an alternative to the conventional superposition of atomic densities (SAD) for initializing self‐consistent field (SCF) iterations. In addition, PARSEC.py uses CuPy to accelerate key computational kernels on GPUs, including diagonalization and Hartree potential updates.

In the following sections, we describe PARSEC.py's theoretical framework, core algorithms, and its integration of ML‐predicted densities as initial guesses to accelerate SCF convergence further. We also present its CuPy‐based GPU acceleration for key computational steps, including diagonalization and Hartree potential updates. Selected molecular examples demonstrate the accuracy and computational efficiency of the framework.

## 2 Real‐Space KS‐DFT Theory

### 2.1 KS Equation

Real‐space KS‐DFT is based on the KS equation [1, 2]:

(1) $$\begin{array}{c} H\phi_{i}(\vec{r})=\left\{-\frac{1}{2}\nabla^{2}+V_{ion}(\vec{r})+V_{H}\left[\rho (\vec{r}),\vec{r}\right]+V_{xc}\left[\rho (\vec{r}),\vec{r}\right]\right\}\phi_{i}(\vec{r})\\ \;=\epsilon_{i}\phi_{i}(\vec{r})\;i=1,2,\text{…} \end{array}$$

where atomic units ($\hbar =m_{e}=e=4\pi \epsilon_{0}=1$) are used and will be used for all formulas throughout the manuscript. $\phi_{i}(\vec{r})$ is the KS orbital/wavefunction/eigenfunction, $\epsilon_{i}$ is the orbital energy/eigenvalue, and $\vec{r}$ is the position in space. $\rho (\vec{r})$ represents the charge density and is defined as:

(2) $$\rho (\vec{r})=2\overset{n_{occ}}{\underset{i=1}{\sum }}f_{i}|\phi_{i}(\vec{r}){|}^{2}$$

where $n_{occ}$ is the number of occupied orbitals, $f_{i}$ is the occupation number ($0\leq f_{i}\leq 1$) of the ith occupied orbital, which can be obtained from the Fermi‐Dirac statistics: [69, 70]

(3) $$\begin{array}{c} f_{i}(\epsilon_{F})=\frac{1}{1+e^{(\epsilon_{i}-\epsilon_{F})/kT}}\;2\overset{n_{occ}}{\underset{i=1}{\sum }}f_{i}(\epsilon_{F})=n_{e} \end{array}$$

where $\epsilon_{F}$ is the Fermi level, k is the Boltzmann constant, T is the absolute temperature, and $n_{e}$ is the number of electrons. The Fermi‐Dirac expression is used here as a numerical smearing scheme for determining orbital occupations during SCF iterations. The total number of electrons remains fixed by the constraint in Equation (3) and is therefore still an integer for each molecular system. For closed‐shell molecules with sizable HOMO‐LUMO gaps, the smearing temperature used in this work, T=500 K ($k_{B}T\approx 0.043$ eV), gives occupations that are effectively integer. Fractional occupations only become significant for near‐degenerate frontier orbitals or open‐shell systems, for which smearing improves the numerical stability of the SCF procedure. The use of LDA may reduce the KS HOMO‐LUMO gap, but the smearing is introduced primarily as a robust numerical occupation scheme rather than as a physical finite‐temperature molecular model.

The Hamiltonian H has four components. $\nabla^{2}$ is the Laplacian. $V_{ion}$ is the ionic potential determined by the ionic cores. $V_{H}$ is the Hartree potential, which is updated through the Poisson equation if an initial value is given:

(4) $$\nabla^{2}V_{H}=-4\pi \rho (\vec{r})$$

$V_{xc}$ is the exchange‐correlation potential, which can be approximated by the local density approximation (LDA) [2], the generalized gradient approximation (GGA) [71], other LDA/GGA variants [72, 73, 74, 75], or higher rungs in Jacob's ladder [76].

### 2.2 PP Theory

In most practical electronic structure calculations, the pseudopotential (PP) approach is preferred over the all‐electron (AE) approach due to its substantial computational advantages. By replacing the strong Coulomb potential of the nucleus and tightly bound core electrons with a smoother effective potential [77, 78, 79], PPs eliminate the need to resolve the rapidly oscillating core wavefunctions, which require extremely fine grids or large basis sets. Since core electrons are usually chemically inert, this approximation introduces only a small loss of accuracy for most material properties. As a result, PPs significantly reduce computational cost while still producing results close to those of the AE counterpart.

Within the PP approximation, one can write the ionic potential $V_{ion}$ in Kleinman–Bylander (KB) form with local and nonlocal parts [80]:

(5) $$\begin{array}{c} V_{ion}(\vec{r})=V_{loc}(\vec{r})+V_{NL}(\vec{r})\\ \;=\underset{a}{\sum }V_{loc}^{a}(r^{a})+\underset{a,nlm}{\sum }\frac{\left|\Delta V_{nl}^{a}(r^{a})\phi_{nlm}^{a}(r^{a})\rangle \langle \phi_{nlm}^{a}(r^{a})\Delta V_{nl}^{a}(r^{a})\right|}{\left\langle \phi_{nlm}^{a}(r^{a})|\Delta V_{nl}^{a}(r^{a})|\phi_{nlm}^{a}(r^{a})\right\rangle }\\ \;r^{a}=|\vec{r}-{\vec{R}}^{a}|\;\Delta V_{nl}^{a}(r)=V_{nl}^{a}(r)-V_{loc}^{a}(r)\\ \;V_{nl}^{a}(r)=V_{loc}^{a}(r)=V_{ion,AE}^{a}(r)\;\text{for}\;r>r_{c}^{a} \end{array}$$

where a labels the atom in the system and ${\vec{R}}^{a}$ is its nucleus position. $\phi_{nlm}^{a}$ is the atomic pseudo‐wavefunction corresponding to quantum numbers n,l,m for the a‐th atom. $V_{nl}^{a}$ is the corresponding PP and $V_{loc}^{a}$ is the local PP chosen from $V_{nl}^{a}$. $r_{c}^{a}$ is the radius of the atomic core region that indicates the range for using atomic PPs to replace the AE core potential of atom $V_{ion,AE}^{a}$. In practice, the choice of $V_{loc}^{a}$ can be arbitrary. For example, choosing either 2s or 2p as the local part is allowed for 2nd row elements. If $V_{2s}^{a}$ is selected as the local PP, then it must be subtracted from $V_{2p}^{a}$ when constructing the nonlocal part. To improve accuracy, one can benchmark the final results among different choices. However, two things need to be emphasized. First, all PPs outside $r_{c}$ are the same, set to the AE potential $V_{ion,AE}^{a}$. This indicates that there is no nonlocal contribution outside $r_{c}^{a}$, which means that the nonlocal potential is a short‐range interaction. Second, the summation of n,l,m should be interpreted as the inclusion of all valence orbitals.

### 2.3 Construction of Initial Hamiltonian in Real‐Space KS‐DFT

#### 2.3.1 Real‐Space Representation

The formulas above are in continuous form. To achieve a real‐space representation, we discretize the simulation domain on a uniform Cartesian grid with spacing h and a total number of points $n=n_{x}\times n_{y}\times n_{z}$, where $n_{x}$, $n_{y}$, $n_{z}$ denote the number of grid points along the x, y, z directions. Let f be a discrete function whose value depends on the position of the grid point. This grid function $f({\vec{r}}_{ijk})$ can then be represented as a vector $\vec{f}\in {\mathbb{R}}^{n}$ by flattening the three‐dimensional (3D) grids. ${\mathbb{R}}^{n}$ stands for the n‐dimensional real vector space. The component in the vector is the function value at the grid points ${\vec{r}}_{ijk}$, with i,j,k denoting the indices along the x,y,z directions. Within this real space representation, the KS orbitals become vectors ${\vec{\phi }}_{i}\in {\mathbb{R}}^{n}$, and the electron density is represented by $\vec{\rho }\in {\mathbb{R}}^{n}$. Although, KS orbitals may be complex (${\vec{\phi }}_{i}\in {\mathbb{C}}^{n}$, ${\mathbb{C}}^{n}$ stands for n‐dimensional complex vector space), in this work, we restrict ourselves to real orbitals. Since, ${\vec{\phi }}_{i}$ is evaluated only on grid points inside the domain, we equivalently extend it to zero outside by imposing a homogeneous Dirichlet boundary conditions. With the real‐space representation above, we move on to discuss the concrete matrix forms for all the terms in the Hamiltonian.

#### 2.3.2 Matrix Form for Laplacian Within FD

**FIGURE 1:**
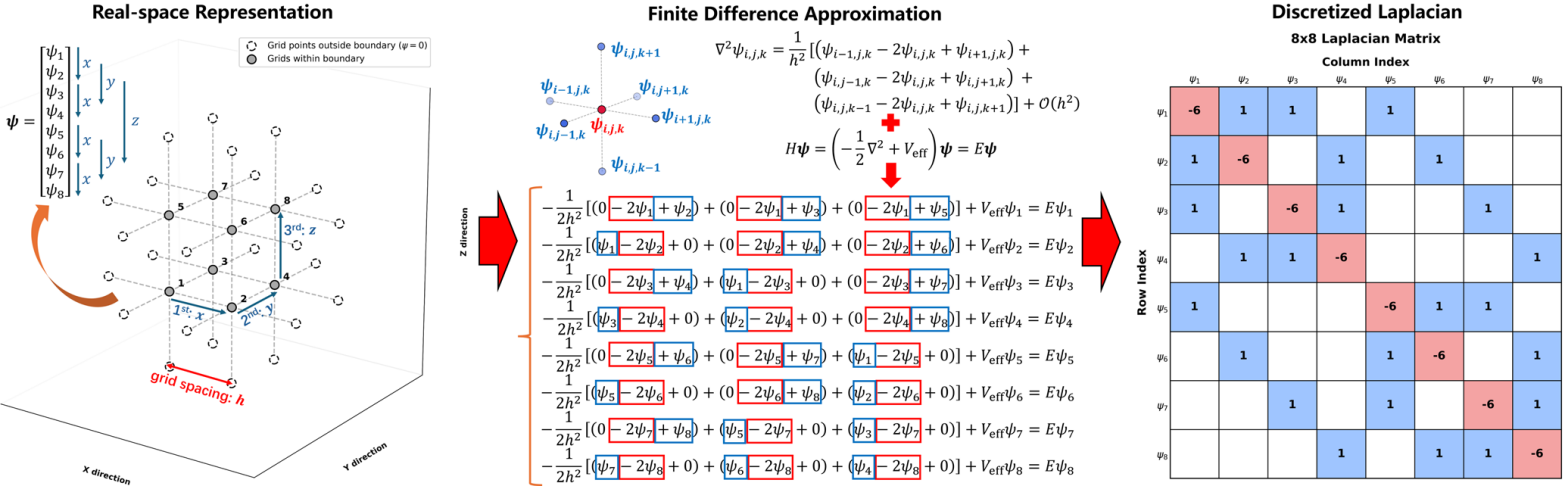
Construction of the Laplacian matrix in the real‐space representation using the FD method with N=1 for the simplest 3D case of eight grid points. The grid points are ordered first along x, then y, and finally z.

The FD method lies at the heart of the real‐space KS‐DFT framework. We can write the 3D Laplacian in terms of the KS orbital values projected on the uniform grids: [10, 11]

(6) $$\begin{array}{c} \nabla^{2}f(x_{i},y_{j},z_{k})=\frac{1}{h^{2}}\left[\overset{N}{\underset{l_{x}=-N}{\sum }}C_{l_{x}}f(x_{i}+l_{x}h,y_{j},z_{k})\right.\\ \;+\overset{N}{\underset{l_{y}=-N}{\sum }}C_{l_{y}}f(x_{i},y_{j}+l_{y}h,z_{k})\\ \;+\left.\overset{N}{\underset{l_{z}=-N}{\sum }}C_{l_{z}}f(x_{i},y_{j},z_{k}+l_{z}h)\right]+𝒪(h^{2N}) \end{array}$$

where N is the order of the expanded terms, $C_{l_{x}}$, $C_{l_{y}}$, and $C_{l_{z}}$ are the coefficients corresponding to the expanded terms, and the Laplacian is expanded based on the grid point $\left(x_{i},y_{j},z_{k}\right)$. The expansion coefficients for the FD expressions up to N=5 are given in Table 1. Figure 1 shows a simple 3D example of how the Laplacian matrix is determined when N=1. We denote the real‐space matrix representation of the negative Laplacian by L.

**TABLE 1:** Expansion coefficients for FD expressions [81].

Order	$C_{0}$	$C_{\pm 1}$	$C_{\pm 2}$	$C_{\pm 3}$	$C_{\pm 4}$	$C_{\pm 5}$
N=1	−2	1				
N=2	−5/2	4/3	−1/12			
N=3	−49/18	3/2	−3/20	1/90		
N=4	−205/72	8/5	−1/5	8/315	−1/560	
N=5	−5269/1800	5/3	−5/21	5/126	−5/1008	1/3150

#### 2.3.3 Diagonal Potential Terms $V_{\mathrm{loc}}$, $V_{H}$, $V_{\mathrm{xc}}$ Initialized by SAD vs. ML Density

PP files, including radial atomic pseudo‐wavefunctions $\phi_{nl}^{a}$ and PPs $V_{nl}^{a}$ for different valence orbitals, are required before the real‐space KS‐DFT calculation. The initial atomic densities $\rho_{0}^{a}$ and the corresponding atomic Hartree potentials $V_{H,0}^{a}$ are also needed. The atomic radial data we use in the calculation are tabulated on an adapted radial grid whose spacing increases progressively with distance from the atomic center, ranging from $10^{-5}$ a.u. near the center to about 1 a.u. at large distances. Spline interpolation is then used to construct a continuous representation for projection onto the chosen real‐space grid domain.

${\vec{V}}_{loc}^{a}$, ${\vec{V}}_{loc}$ is then built using the superposition shown in Equation (5). When using the SAD method for the initial guess, the initial density ${\vec{\rho }}_{0}^{SAD}$ and the initial Hartree potential ${\vec{V}}_{H,0}^{SAD}$ at grid point ${\vec{r}}_{ijk}$ are also obtained through superposition. In contrast, the ML method uses the predicted density data ${\vec{\rho }}_{0}^{ML}$ directly and computes the Hartree potential ${\vec{V}}_{H,0}^{ML}$ from Equation (4). Within local or semi‐local density functional frameworks like LDA or GGA, the initial exchange‐correlation potential ${\vec{V}}_{xc,0}$ can then be calculated as long as the density and the analytical form of the density functional are available. Table 2 lists how we compute the quantities related to the diagonal matrix elements in the Hamiltonian.

**TABLE 2:** ${\vec{V}}_{loc}$, ${\vec{\rho }}_{0}$, ${\vec{V}}_{H,0}$ and ${\vec{V}}_{xc,0}$ at a specific grid point ${\vec{r}}_{ijk}$ for different methods.

	SAD	ML
$V_{\mathrm{loc}}^{\mathrm{ijk}}$	$\sum_{a}V_{loc}^{a}\;\left(\left|{\vec{r}}_{ijk}-{\vec{R}}^{a}\right|\right)$	$\sum_{a}V_{loc}^{a}\;\left(\left|{\vec{r}}_{ijk}-{\vec{R}}^{a}\right|\right)$
$\rho_{0}^{\mathrm{ijk}}$	$\rho_{0}^{SAD}=\sum_{a}\rho_{0}^{a}\;\left(\left|{\vec{r}}_{ijk}-{\vec{R}}^{a}\right|\right)$	$\rho_{0}^{ML}({\vec{r}}_{ijk})$
$V_{H,0}^{\mathrm{ijk}}$	$V_{H,0}^{SAD}=\sum_{a}V_{H,0}^{a}\;\left(\left|{\vec{r}}_{ijk}-{\vec{R}}^{a}\right|\right)$	$V_{H,0}^{ML}({\vec{r}}_{ijk})$ ($\nabla^{2}V_{H,0}^{ML}=-4\pi \rho_{0}^{ML}$)
$V_{\mathrm{xc},0}^{\mathrm{ijk}}$	$V_{xc}[\rho_{0}^{SAD}({\vec{r}}_{ijk})]$	$V_{xc}[\rho_{0}^{ML}({\vec{r}}_{ijk})]$

*Note:* The superscript i,j,k represents the value for the grid point at ${\vec{r}}_{ijk}$. It can also be viewed as the index of the component in the vector when real‐space representation is used.

#### 2.3.4 Off‐Diagonal Matrix Elements From the Nonlocal Ionic Potential: $V_{\mathrm{NL}}$

**FIGURE 2:**
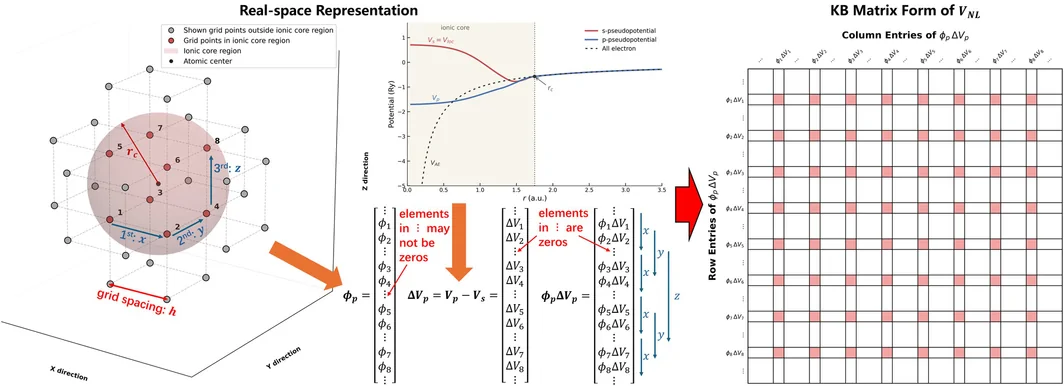
Construction of nonlocal ionic potential matrix in the real‐space representation using KB form for the p orbital in the single‐atom case of eight grid points. The grid points are ordered first along x, then y, and finally z.

With the real‐space representations for ${\vec{\phi }}_{nlm}^{a}$ and ${\vec{V}}_{nl}^{a}$, we can write the nonlocal ionic potential in Equation (5) as a matrix with the following format:

(7) $$\begin{array}{c} \;V_{\mathrm{NL}}=\underset{a,nlm}{\sum }\frac{1}{D_{nl}^{a}}\left({\vec{\phi }}_{nlm}^{a}⊙\Delta {\vec{V}}_{nl}^{a}\right)⊗\left({\vec{\phi }}_{nlm}^{a}⊙\Delta {\vec{V}}_{nl}^{a}\right)\\ \Delta {\vec{V}}_{nl}^{a}={\vec{V}}_{nl}^{a}-{\vec{V}}_{loc}^{a}\\ \;V_{nl}^{a,ijk}=V_{loc}^{a,ijk}=V_{ion,AE}^{a,ijk}\;\text{for}\;|{\vec{r}}_{ijk}-{\vec{R}}^{a}|>r_{c}^{a} \end{array}$$

where $D_{nl}^{a}$ is just the dominator of the nonlocal term in Equation (5). It is written as a single scalar here since we can actually pre‐store it in the PP files for each atom. ⊙ and ⊗ represent the element‐wise product and the outer product, respectively. One usually only stores the radial part of pseudo‐wavefunctions ${\vec{\phi }}_{nl}^{a}$ and projects it in the direction the orbital orients to obtain the angular/direction‐dependent part ${\vec{\phi }}_{nlm}^{a}$. The following equations use p orbitals as an example:

(8) $${\vec{\phi }}_{p_{\alpha }}^{a}=\frac{\left|\alpha_{ijk}-\alpha^{a}\right|}{\left|{\vec{r}}_{ijk}-{\vec{R}}^{a}\right|}{\vec{\phi }}_{p}^{a}\;\alpha =x,y,z$$

where $x_{ijk},y_{ijk},z_{ijk}$ are the x,y,z components of ${\vec{r}}_{ijk}$ and $x^{a},y^{a},z^{a}$ are the x,y,z components of ${\vec{R}}^{a}$. Considering the short‐range character of the nonlocal potential, $V_{\mathrm{NL}}$ is then a sparse matrix. Figure 2 shows a simple 3D example of how $V_{\mathrm{NL}}$ is determined.

### 2.4 Diagonalization of the Hamiltonian

Having all terms expressed in real‐space, we can now write the matrix form of our Hamiltonian.

(9) $$H=\frac{1}{2}L+V_{NL}+\mathrm{diag}\left({\vec{V}}_{loc}+{\vec{V}}_{H,0}+{\vec{V}}_{xc,0}\right).$$

The Hamiltonian matrix expressed in real space has a large dimension, particularly for large computational domains and small grid spacing h. For example, if the box length is 10 Å (corresponding to a radius of 5 Å) and the grid spacing is h=0.1 Å, the system contains $100^{3}=10^{6}$ grid points, resulting in a Hamiltonian matrix of dimension $10^{6}\times 10^{6}$. As a result, the diagonalization step becomes extremely time‐consuming. However, in practice, only the occupied states and a small number of low‐lying virtual states are of interest. This motivates the use of subspace projection techniques to filter out irrelevant portions of the spectrum. Nevertheless, a reckless application of subspace filtering can be inaccurate and may discard important information from the original Hamiltonian. It is of great significance to carefully estimate the extreme eigenvalues of the matrix, select the filtering polynomials, and set the upper and lower bounds to apply the filtering polynomials for subspace filtering. We first introduce the generic algorithmic tools needed, then list specialized diagonalization methods for handling the gigantic real‐space‐represented Hamiltonian.

#### 2.4.1 Numerical Techniques for Diagonalization

##### 2.4.1.1 Lanczos Procedure

The Lanczos procedure iteratively projects a large Hermitian matrix onto a low‐dimensional subspace to extract spectral information [82, 83]. Starting from a single vector, it builds an orthonormal basis $V_{m}$, whose columns are referred to as Lanczos vectors, through m steps of successive matrix applications based on a three‐term recurrence, producing a tridiagonal representation $T_{m}=V_{m}^{†}HV_{m}$. The resulting reduced eigenproblem for $T_{m}$ is inexpensive to solve. Its eigenvectors $Q_{m}$ and eigenvalues $\Lambda_{m}$ provide approximations to the extremal eigenpairs of the original matrix H in the form $\left\{V_{m}Q_{m},\Lambda_{m}\right\}$ as the subspace dimension m increases. For clarity, the detailed algorithms and formulations are provided in the Supporting Information (Section S1).

In exact arithmetic, the orthogonality of $V_{m}$ guarantees stability; in finite precision, the loss of orthogonality can cause spurious effects. Full reorthogonalization at each step restores stability but is costly for large m [84]. A more efficient approach truncates $Q_{m}$ to $m_{0}$ columns after m iterations, reorthogonalizes $V_{m}Q_{m\times m_{0}}$, and solves the projected eigenproblem in this reduced subspace [85, 86]. Convergence can be assessed by monitoring the sum of eigenvalues rather than fixing m. These strategies balance robustness, efficiency, and memory usage, making the Lanczos method effective for large‐scale eigenvalue problems [87].

##### 2.4.1.2 Chebyshev Subspace Filtering

Suppose we have the following set of real‐space‐represented wavefunctions:

(10) $$\Phi =[{\vec{\phi }}_{1},{\vec{\phi }}_{2},\text{⋯}\;,{\vec{\phi }}_{m}].$$

We can then construct a projection operator using a Hamiltonian form of polynomials and apply it to Φ:

(11) $$\begin{array}{c} \begin{array}{ccc} P(H)\Phi & \;=\left[\begin{array}{ccc} \text{⋯} & \left(\overset{M}{\underset{n=1}{\sum }}a_{n}H^{n}\right){\vec{\phi }}_{l} & \text{⋯} \end{array}\right] & \\ & \;=\left[\begin{array}{ccc} \text{⋯} & \left(\overset{M}{\underset{n=1}{\sum }}a_{n}\varepsilon_{l}^{n}\right){\vec{\phi }}_{l} & \text{⋯} \end{array}\right]=\left[\begin{array}{ccc} \text{⋯} & P(\varepsilon_{l}){\vec{\phi }}_{l} & \text{⋯} \end{array}\right] & \end{array} \end{array}$$

where M denotes the polynomial degree. If the factor $P(\varepsilon_{l})$ is close to zero for eigenvalues above a specific threshold and remains large for the others, we then “filter out” the components that have higher energies and only keep the states we are interested in.

Chebyshev polynomial filtering is a technique to accelerate subspace iteration by selectively amplifying spectral components associated with targeted eigenvalues: [15, 88, 89, 90, 91]

(12) $$\begin{array}{c} P_{0}(x)=1,P_{1}(x)=x,\\ P_{n}(x)=2xP_{n-1}(x)-P_{n-2}(x)\;n\geq 2.\; \end{array}$$

The Chebyshev polynomials stay close to zero within [−1,1], while having large values outside. These polynomials are easy to expand to higher orders using the recursion relation Equation (12).

The next step is to map the unwanted states in the energy spectrum to the range [−1,1]. Suppose that the full spectrum of the Hamiltonian H, denoted as σ(H), spans within $\left[a_{0},b\right]$, and we only want the states in the range $\left[a_{0},a](a_{0}<a<b\right)$. Then, we need to map [a,b] to [−1,1].

(13) $$\begin{array}{c} \Phi^{\prime }=P\left(\frac{H-c}{e}\right)\Phi \;\text{or}\;\frac{P\left(\frac{H-c}{e}\right)}{P\left(\frac{a_{0}-c}{e}\right)}\Phi ,\\ \;c=\frac{a+b}{2},e=\frac{b-a}{2} \end{array}$$

At high polynomial degrees, exponential growth outside [−1,1] can cause numerical instability; this is mitigated by normalized Chebyshev filtering, as in the second mapping in Equation (13), which scales the polynomial at the smallest eigenvalue to preserve spectral selectivity and ensure stability.

In practice, the lower bound a is set to the largest eigenvalue from the previous iteration, while the upper bound b is estimated using Lanczos procedures. As Chebyshev filtering is non‐unitary, the filtered wavefunctions are reorthogonalized before solving the subspace eigenproblem for H.

##### 2.4.1.3 Orthonormalization

Both the Lanczos procedure and Chebyshev subspace filtering require reorthogonalization of the basis. A common orthonormalization method is the Gram–Schmidt process [92]. It constructs an orthonormal basis by sequentially orthogonalizing each vector against the previously obtained basis vectors through projection removal. An efficient alternative is the Cholesky‐QR algorithm, which constructs an orthonormal basis by forming a matrix from the original vectors, computing a triangular factor from their inner products, and using it to normalize and decorrelate the basis. A more numerically stable alternative is the Householder QR algorithm. It produces an orthonormal basis via a sequence of orthogonal reflections that systematically eliminate subdiagonal components, yielding a numerically stable factorization. For clarity, the detailed algorithms and formulations are provided in the Supporting Information (Section S2).

These orthogonalization methods balance accuracy and efficiency differently. Householder QR provides backward stability and near machine‐precision orthogonality, regardless of the conditioning of the basis. Classical Gram–Schmidt is simple but unstable, whereas modified Gram–Schmidt improves robustness at the cost of additional synchronization. Cholesky‐QR exploits factorization to enable efficient implementations and strong scalability, but it amplifies conditioning and may lose orthogonality in ill‐conditioned bases. Consequently, Householder QR is preferred for accuracy, whereas Cholesky‐QR and stabilized Gram–Schmidt are preferred in large‐scale block methods with well‐conditioned input [93, 94, 95]. We use SciPy's default Householder QR implementation in our Python code [25].

##### 2.4.1.4 Rayleigh–Ritz Step

A standard approach for solving the subspace eigenproblem of H, both during the truncated Lanczos procedure and after Chebyshev filtering, is the Rayleigh–Ritz (RR) method [96, 97, 98].

Given a Hamiltonian matrix $H\in {\mathbb{C}}^{n\times n}$, we project it onto an m‐dimensional subspace $H^{\prime }\in {\mathbb{C}}^{m\times m}$ (m<n) spanned by an orthonormal basis $W\in {\mathbb{C}}^{n\times m}$. Solving the resulting subspace eigenproblem yields eigenpairs $\left\{\Psi^{\prime },\varepsilon \right\}$ that approximate the eigenpairs {Ψ,ε} of H.

(14) $$\begin{array}{c} \begin{array}{c} \;H^{\prime }=W^{†}HW\to H^{\prime }\Psi^{\prime }=\Psi^{\prime }\varepsilon \overset{\text{solve subspace}}{\underset{\text{eigenproblem}}{\to }}\\ H(W\Psi^{\prime })=(W\Psi^{\prime })\varepsilon \overset{\text{full space}}{\underset{\text{eigenpairs}}{\to }}\{\Psi =W\Psi^{\prime },\varepsilon \}. \end{array} \end{array}$$

If W contains k(≤m) vectors close to the eigenvectors of H, the RR method produces k accurate Ritz vectors, whose precision can be assessed using the residual norm $\left\|(H-\varepsilon )W\Psi^{\prime }\right\|$.

#### 2.4.2 Diagonalization Methods

##### 2.4.2.1 Lanczos‐RR (LRR) Process

In an m‐step Lanczos procedure, as mentioned before, instead of reorthogonalizing Lanczos vectors at each iteration, one may construct $V_{m}$ in finite precision, solve the associated eigenproblem for the tridiagonal matrix $T_{m}$, and truncate the eigenvector matrix $Q_{m}$ to $m_{0}$ columns. The corresponding Ritz vectors $V_{m}Q_{m\times m_{0}}$ are then reorthogonalized, and the eigenproblem of H is solved in this subspace via the RR method, retaining the required eigenpairs. The eigenvalues from the Lanczos and RR steps are noted as $\Lambda_{m}$ and $\varepsilon_{m_{0}}$, respectively. Let n denote the dimension of H, $n_{e}$ the total number of valence electrons, and $n_{ev}$ the number of requested eigenpairs. Since, the eigenproblem of H is solved indirectly, the computed eigenpairs are approximate. To ensure the desired ones are captured, we may need to extract a larger number of eigenpairs $n_{ev}^{\prime }(\geq n_{ev}$). The detailed procedure is given in Equation (15) with explicit dimension subscripts; for diagonal eigenvalue matrices, only a single dimension is indicated. We refer to the procedure as the LRR process.
(15)

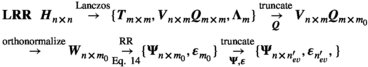



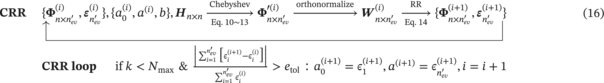


##### 2.4.2.2 Chebyshev‐RR (CRR) Process

Starting from the grid‐based wavefunction matrix $\Phi_{n\times n_{ev}^{\prime }}$, which contains the target eigenvectors, we integrate Chebyshev subspace filtering, orthonormalization, and the subsequent RR step into a unified procedure termed the CRR process. Using the notation introduced above, with prescribed bounds a, b and scaling eigenvalue $a_{0}$, the CRR process is illustrated in Equation (16).

In the initial diagonalization, a single CRR may not yield sufficiently accurate eigenpairs; thus, CRR iterations are repeated until the maximum number of iterations, $N_{\max}$, is reached or the sum of eigenvalues converges within the relative tolerance $e_{\text{tol}}$. We denote the iteration index by the superscript (i). The upper bound b remains fixed. The lower bound a and the scaling parameter $a_{0}$ for normalized Chebyshev filtering are updated to the largest and smallest Ritz values, $\epsilon_{n_{ev}^{\prime }}$ and $\epsilon_{1}$, respectively, from the previous iteration. We refer to this iterative scheme as the CRR loop. The polynomial degree for Chebyshev filtering is set to M=10.

##### 2.4.2.3 Implemented Diagonalization Methods

Based on the two processes above, we implemented four types of diagonalization methods.

The first method performs a long LRR process (Equation (15)) and is denoted “Lanczos”; its final eigenpairs approximate those of H.

The second approach, Chebyshev subspace iteration (“ChSupsb”), begins with a long LRR process to estimate the initial eigenpairs and spectral bounds carefully. Then it performs a long CRR loop (Equation (16)) with normalized Chebyshev filtering (Equation (13)). If convergence is achieved or the maximum iteration is reached, the resulting eigenpairs are taken as approximations to those of H.

The third option, first‐step diagonalization by subspace filtering (“FirstFilt”), starts with a short Lanczos procedure (m=6∼10) to obtain rough initial upper and lower bounds [99, 100]. The initial eigenvectors are randomly initialized, followed by a short CRR loop with direct Chebyshev filtering (Equation (13)). If the loop converges or reaches the maximum iteration, the resulting eigenpairs approximate those of H.

The final choice, Chebyshev‐filtered subspace iteration (“CheFSI”), is applied after the first SCF iteration [90]. It initializes eigenpairs from the previous SCF step and roughly estimates spectral bounds via a short Lanczos procedure (m=6). The bounds and scaling parameter are set as in the ChSupsb approach. A single CRR step (Equation (16)) with normalized Chebyshev filtering is then performed, yielding updated eigenpairs to approximate those of H.

Based on the algorithms underlying these methods, we summarize their respective advantages and limitations as follows. The Lanczos approach avoids the construction of bounds and the use of Chebyshev filtering, making it a robust diagonalization method; however, it can be slow for large m. ChSubsp performs a long LRR run to obtain reliable spectral bounds and a high‐quality initial subspace, combined with scaled Chebyshev filtering for numerical stability; it is the most robust option for the first SCF step but incurs high computational cost due to the long LRR phase and multiple CRR iterations. FirstFilt relies on a short Lanczos run with averaged upper‐bound estimates and an unscaled Chebyshev filter, offering low memory usage and high speed, but at the expense of reduced robustness and greater sensitivity to parameter choices. CheFSI also uses a short Lanczos run for bound estimation and reuses previous Ritz values, together with scaled Chebyshev filtering, resulting in minimal overhead and the fastest performance per SCF step; however, it depends on a good prior subspace and accurate bounds, and is therefore unsuitable for the initial diagonalization.

In light of the respective advantages and limitations discussed above, we adopt Lanczos, ChSubsp, or FirstFilt for Hamiltonian diagonalization in the initial SCF iteration and employ CheFSI for subsequent SCF iterations until convergence is achieved. Table 3 summarizes the characteristics, pros, and cons of all methods, while Table S1 summarizes the default parameter settings, iteration pattern, and usage of them. The Lanczos method can be employed for all SCF iterations; however, it is computationally expensive.

**TABLE 3:** Characteristics, pros, and cons for the diagonalization methods.

Method	Lanczos	ChSubsp	FirstFilt	CheFSI
LRR or Lanczos steps	Long	Long	Short	Short
Initial wavefunction	N/A	From LRR	Randomly initialized	From previous SCF iteration
Filter bounds	N/A	Carefully estimated	Roughly estimated	Roughly estimated
CRR steps	N/A	Long	Short	1
Filter type	N/A	Normalized Chebyshev	Chebyshev	Normalized Chebyshev
Usage	First SCF	First SCF	First SCF	After first SCF
Pros and Cons	No bound/filtering; robust cold‐start; slow for long steps.	suitable bounds; reliable subspace; numerically stable; robust but expensive	low memory usage; high speed; reduced robustness; sensitive to parameters.	minimal overhead; fastest performance; sensitive to bounds and prior subspace; unsuitable for initial diagonalization.

### 2.5 SCF Iteration

#### 2.5.1 Update Occupation Number

After diagonalizing the Hamiltonian H, we obtain the eigenpairs. Since the Fermi level $\epsilon_{F}$ is unknown, the occupation numbers $\left\{f_{i}(\epsilon_{F})\right\}$ arecomputed from Equation (3) using the bisection method [101]. For clarity, the detailed algorithms and formulations are provided in the Supporting Information (Section S4).

#### 2.5.2 Update Charge Density

When we obtain the eigenpairs $\left\{\Phi_{n\times n_{ev}},\varepsilon_{n_{ev}}\right\}$ and the occupation vector $\vec{f}={\left[f_{1},\text{…},f_{n_{ev}}\right]}^{T}$, we can calculate the new charge density through Equation (2) in a real space representation:

(17) $$\vec{\rho }=2|\Phi_{n\times n_{ev}}{|}^{2}\cdot \vec{f}$$

Here, $|\Phi_{n\times n_{ev}}{|}^{2}$ denotes a matrix whose entries are the squared moduli of the corresponding elements of $\Phi_{n\times n_{ev}}$,

(18) $$\begin{array}{c} {\left(|\Phi_{n\times n_{ev}}{|}^{2}\right)}_{ij}=|{\left(\Phi_{n\times n_{ev}}\right)}_{ij}{|}^{2} \end{array}$$

where i and j index the row and column, respectively.

#### 2.5.3 Update Hamiltonian

In the Hamiltonian H (Equation (9)), the negative Laplacian L is fixed once the real‐space grid is defined. The pseudopotentials, both the nonlocal term $V_{NL}$ and the local term ${\vec{V}}_{loc}$, are also fixed, as they depend only on ionic cores. Only the Hartree potential ${\vec{V}}_{H}$ and the exchange‐correlation potential ${\vec{V}}_{xc}$ vary with the charge density. ${\vec{V}}_{xc}$ is straightforward to update given the density functional, while ${\vec{V}}_{H}$ requires solving the Poisson equation (Equation (4)) in real space. In this work, ${\vec{V}}_{H}$ is updated indirectly via a correction based on the previous SCF iteration.

We first decompose $\rho ,V_{H}$ into reference terms $\rho^{0},V_{H}^{0}$ and correction terms $\Delta \rho ,\Delta V_{H}$ in continuous form:

(19) $$\rho (\vec{r})=\rho^{0}(\vec{r})+\Delta \rho (\vec{r})\;V_{H}(\vec{r})=V_{H}^{0}(\vec{r})+\Delta V_{H}(\vec{r}),$$

Since $\rho ,V_{H}$ satisfy Equation (4) in both the previous and current SCF iterations, $\Delta V_{H}$ and Δρ obey another Poisson equation: 
(20)

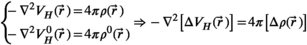

We solve for $\Delta V_{H}$ and recover the total potential as $V_{H}=V_{H}^{0}+\Delta V_{H}$. This reformulation is numerically more stable, as the smoother and typically smaller right‐hand side improves conditioning during SCF iterations.

We prove that the operator $-\nabla^{2}$ is positive definite in SI, and the FD discretization preserves this property; therefore, the Laplacian matrix L is symmetric positive definite, which means that the conjugate gradient (CG) method [102] is applicable to solve Equation (20) in its real space form. (see Figure S2 for detailed CG procedures).

With the updated ${\vec{V}}_{H}$ and ${\vec{V}}_{xc}$, a new effective potential, and thus a new Hamiltonian, is obtained, which could in principle replace the previous one directly. However, in real‐space DFT implementations with Poisson solvers, the SC mapping is not inherently stable: small variations in $\vec{\rho }$ can induce large changes in ${\vec{V}}_{H}$, particularly for long‐wavelength components. Consequently, direct replacement often leads to oscillations or divergence of the SCF cycle (charge sloshing). Mixing stabilizes this feedback process by moderating the potential update and incorporating information from previous iterations; advanced schemes further approximate the system's response to accelerate convergence. In this work, we implement the mixing strategies from Haw‐ren et al. [103] and adopt the Type‐I method [103] described in their paper as the default approach.

#### 2.5.4 Update Energies

With the eigenvalue vector $\vec{\varepsilon }=\text{diag}(\varepsilon_{n_{ev}})$, occupation vector $\vec{f}$, real‐space density $\vec{\rho }$, and real‐space potentials ${\vec{V}}_{H},{\vec{V}}_{xc}$, individual energy contributions can be calculated. Given the grid spacing h and exchange‐correlation energy density ${\vec{\varepsilon }}_{xc}$, the Hartree energy $E_{H}$, and exchange‐correlation energy $E_{xc}$ are obtained as follows:

(21) $$\begin{array}{c} E_{H}=\frac{1}{2}\int \rho (\vec{r})V_{H}(\vec{r})\;d\vec{r}=\frac{1}{2}h^{3}{\vec{\rho }}^{T}\vec{V_{H}}\\ E_{xc}=\int \rho (\vec{r})\varepsilon_{xc}(\vec{r})\;d\vec{r}=h^{3}{\vec{\rho }}^{T}\vec{\varepsilon_{xc}} \end{array}$$

From the KS formalism, the total electronic energy $E_{elec}$ and the total energy $E_{tot}$ are given by

(22) $$E_{elec}=2{\vec{f}}^{T}\vec{\varepsilon }-E_{H}+E_{xc}-{\vec{\rho }}^{T}{\vec{V}}_{H}\;E_{tot}=E_{elec}+E_{nuc}$$

where $E_{nuc}$ denotes the nuclear–nuclear repulsion energy.

#### 2.5.5 The SCF Loop

**FIGURE 3:**
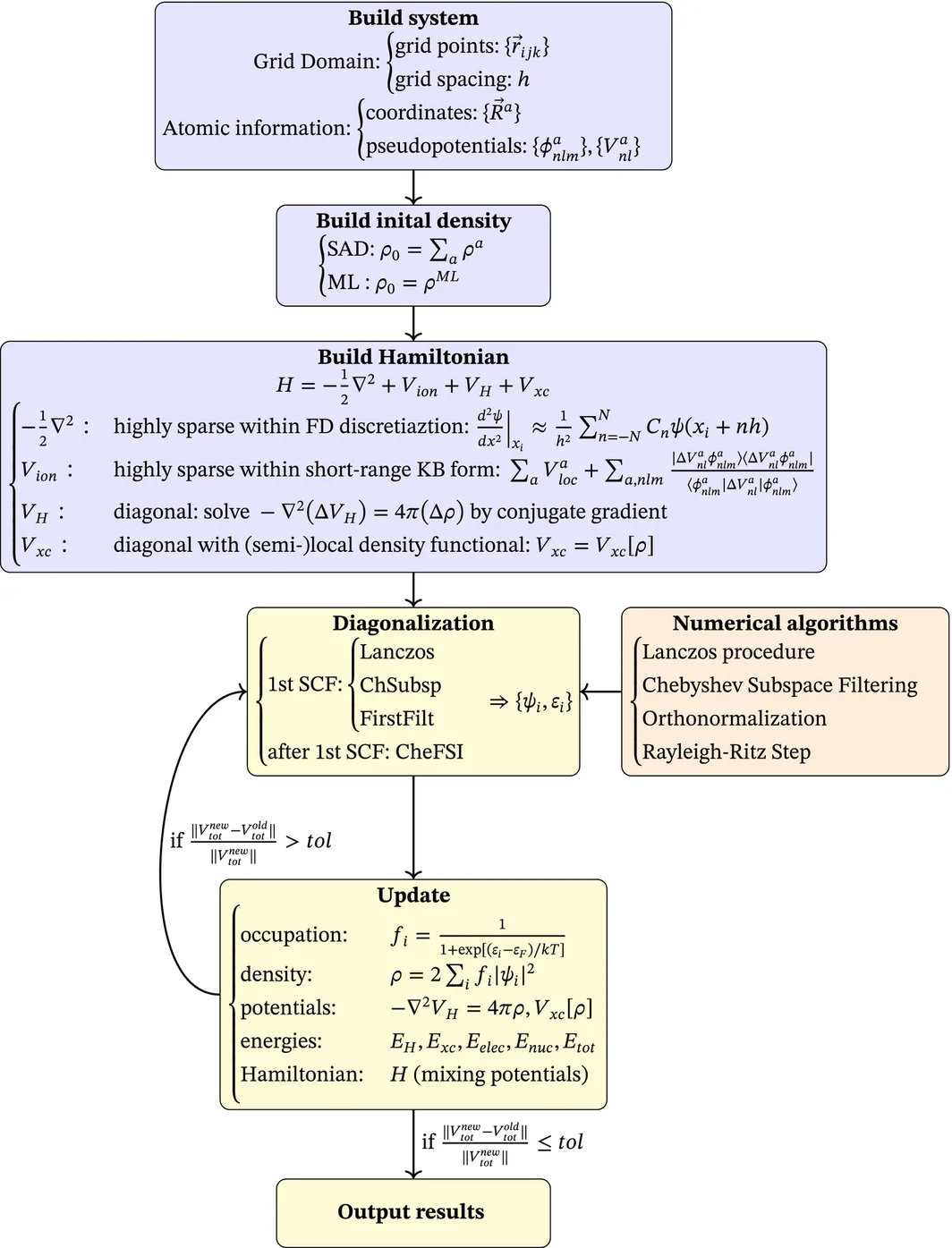
Illustration of the SCF loop implemented in PARSEC.py.

After updating the Hamiltonian H, we can perform the next diagonalization again to obtain new quantities, and so on. Here we illustrate the complete SCF algorithm in Figure 3. The default convergence criterion of the SCF loop is that the relative difference of the effective potential $\left\|V_{tot}^{new}-V_{tot}^{old}\|/\|V_{tot}^{new}\right\|$ should be smaller than $tol=2\times 10^{-4}$. We adopt the Frobenius norm here for ‖·‖.

## 3 Results and Discussions

We now present results from real‐space KS‐DFT calculations on a set of small molecules, focusing on a comparison between SAD and ML densities as initial guesses. We begin by introducing the structure of the input and output files, along with a user interface for generating input files. Before comparing different initial‐density strategies, we validate PARSEC.py against the original PARSEC code for the same molecular set; as summarized in the Supporting Information, the electronic energies per atom and HOMO‐LUMO gaps agree closely, with mean absolute differences of 0.022eV/atom and 0.017eV, respectively. We then compare computational results for the selected molecules using different diagonalization methods and initial density guesses. The comparison includes the number of SCF iterations, the deviation between the initial and converged electron densities, the differences between the initial and converged Hartree ($E_{H}$) and exchange‐correlation ($E_{xc}$) energies, and the difference in converged energy per atom between the SAD and ML approaches.

### 3.1 Input and Output Structures

**FIGURE 4:**
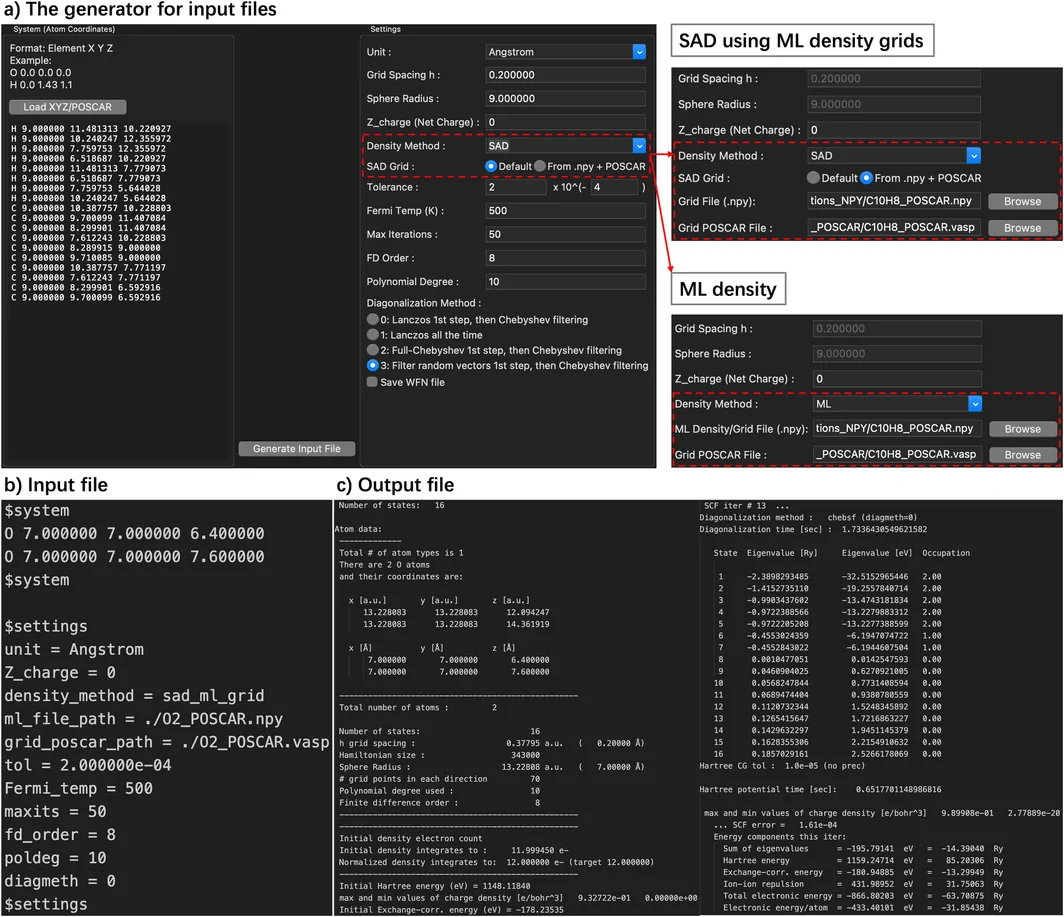
(a) GUI for generating input files with different initial‐density guess settings. (b) The input file for the oxygen molecule. (c) The corresponding output file: left—printout with initial settings; right—printout for one SCF iteration.

We first demonstrate how users can generate a typical input file using the GUI shown in Figure 4a. The left panel displays the atomic coordinates of the system, and the generator supports both XYZ and POSCAR file formats; the right panel lists the parameters for performing real‐space KS‐DFT calculations. The default unit for atomic positions, grid spacing, and radius is angstrom, though it can also be set to Bohr. The default initial density method is SAD, and users can define the grid structure by adjusting the grid spacing and radius. For ML density, the code accepts an NPY file containing the number of grid points in each direction along with the corresponding density values and requires a POSCAR file to determine the simulation box length; the grid spacing and radius are then computed automatically from these two files. To facilitate a direct comparison between SAD and ML initial guesses on the same grid, users need to also adopt the ML grid structure for SAD calculations. The remaining default settings are a relative SCF tolerance of $\|V_{tot}^{new}-V_{tot}^{old}\|/\|V_{tot}^{new}\|\leq 2\times 10^{-4}$, a Fermi temperature of 500 K, a maximum of 50 SCF iterations, an FD order of 8, and a Chebyshev polynomial degree of 10. For the exchange‐correlation potential, we use LDA with Ceperley–Alder parameters [104, 105].

Figure 4b and c presents example input and output files for the oxygen molecule. As described above, the input file consists of two sections: a system section containing the atomic coordinates and a settings section specifying the computational parameters. The parameter order is consistent with that in the generator interface, making the mapping between the GUI and the input file straightforward. The output file is divided into two main parts: the first part prints the initial information, including the atomic coordinates, the number of states used for SCF reporting, the grid structure and Hamiltonian size, the polynomial degree, the FD order, validation of the number of valence electrons, the maximum and minimum values of the charge density, and the initial Hartree and exchange‐correlation energies; the second part reports the SCF iterations, detailing the diagonalization method and timing, eigenvalues and occupations, Hartree potential tolerance and timing, extreme values of the charge density, SCF error, and the individual energy components.

### 3.2 Survey of ML Charge Density Models

SCF calculations within KS‐DFT require an initial guess of the electron density to initiate the iterative procedure. The SAD method is a commonly used initialization strategy [106, 107], but a more accurate initial density can significantly reduce the number of iterations and improve computational efficiency. With the rapid development of ML in electronic‐structure theory [51, 52, 58, 108, 109, 110], ML‐predicted charge densities have emerged as promising alternatives to conventional SAD initialization.

Current ML approaches for charge‐density prediction can be broadly classified into grid/probe‐based methods [61, 63, 111, 112] and orbital/basis‐set‐based methods [62, 64, 113, 114, 115]. Grid‐based methods directly predict the density on discretized 3D real‐space grids by introducing probe nodes and propagating information between atoms and probes via neural networks. This representation is highly flexible and independent of predefined basis functions, enabling accurate modeling of complex spatial features. Still, it can be computationally demanding due to the large number of grid points involved. In contrast, orbital‐based methods represent the density as an expansion in predefined basis functions, such as GTOs combined with spherical harmonics, and predict the associated expansion coefficients. This coefficient‐based representation is more compact and computationally efficient, although the expressivity of the chosen basis limits its accuracy.

As representative examples, we consider ChargE3Net [63] for the grid‐based approach and SCDP [64] for the orbital‐based approach. ChargE3Net employs an E(3)‐equivariant graph neural network to predict real‐space densities via probe‐based message passing. At the same time, SCDP expands density in GTOs and enhances expressivity by incorporating virtual orbitals at interatomic positions, even‐tempered bases, and learnable scaling factors. We select these two methods for their strong performance in their respective categories.

### 3.3 Comparison of SAD and ML Initial Density Guesses for SCF Calculations

We now evaluate whether the ML density can provide a better initial guess and potentially enable an accurate direct prediction of the converged final density. To this end, we employ two ML models–ChargE3Net (denoted as CE3N) and SCDP–both pretrained on the QM9 dataset [61, 116, 117], and compare them on 21 selected small molecules. Because QM9 contains only molecules composed of H, C, N, O, and F atoms, we select 21 small molecules containing these elements exclusively to ensure reliable model predictions. The geometries for nondiatomic molecules (C_10_H_8_, C_2_H_2_, C_2_H_4_, C_2_H_6_, C_3_H_8_O, C_6_H_6_, CH_3_CH_2_CH_2_OH, CH_3_CH_2_OCH_3_, CH_3_CH_2_OH, CH_3_CHOHCH_3_, CH_3_CN, CH_4_, CO_2_, and H_2_O) come from the website [118]. For diatomic molecules (H_2_, HF, CO, F_2_, N_2_, NO, O_2_) the geometries come from the quick optimization by the universal force field within Avogadro [119]. For consistency, the grid spacing is set to 0.2 Å, and an integer sphere radius is used to ensure that the minimum distance between any atom and the boundary is at least 5 Å. This boundary setting was chosen based on a box‐size convergence test for the largest molecule in the benchmark set, ${\text{C}}_{10}{\text{H}}_{8}$, as summarized in the Supporting Information. The test shows that the electronic energy per atom and HOMO‐LUMO gap are sensitive to insufficient vacuum space but converge once the boundary is sufficiently far from the molecule. Thus, the 5 Åminimum atom‐boundary distance used here is sufficient for the algorithmic comparisons. The selected molecules and their corresponding sphere radii or half‐box lengths are listed in Table 4.

**TABLE 4:** Selected molecules and the sphere radii adopted for real space domain.

Molecule	Sphere radius	Molecule	Sphere radius	Molecule	Sphere radius
${\text{C}}_{10}{\text{H}}_{8}$	9 Å	$\text{C}{\text{H}}_{3}\text{C}{\text{H}}_{2}\text{OC}{\text{H}}_{3}$	9 Å	${\text{F}}_{2}$	7 Å
${\text{C}}_{2}{\text{H}}_{2}$	7 Å	$\text{C}{\text{H}}_{3}\text{C}{\text{H}}_{2}\text{OH}$	7 Å	${\text{H}}_{2}$	7 Å
${\text{C}}_{2}{\text{H}}_{4}$	7 Å	$\text{C}{\text{H}}_{3}\text{CHOHC}{\text{H}}_{3}$	9 Å	${\text{H}}_{2}\text{O}$	7 Å
${\text{C}}_{2}{\text{H}}_{6}$	7 Å	$\text{C}{\text{H}}_{3}\text{CN}$	7 Å	HF	7 Å
${\text{C}}_{3}{\text{H}}_{8}\text{O}$	9 Å	$\text{C}{\text{H}}_{4}$	7 Å	${\text{N}}_{2}$	7 Å
${\text{C}}_{6}{\text{H}}_{6}$	9 Å	CO	7 Å	NO	7 Å
$\text{C}{\text{H}}_{3}\text{C}{\text{H}}_{2}\text{C}{\text{H}}_{2}\text{OH}$	9 Å	$\text{C}{\text{O}}_{2}$	7 Å	${\text{O}}_{2}$	7 Å

*Note:* The grid spacing is 0.2 Å.

#### 3.3.1 The Acceleration of SCF Iterations

**FIGURE 5:**
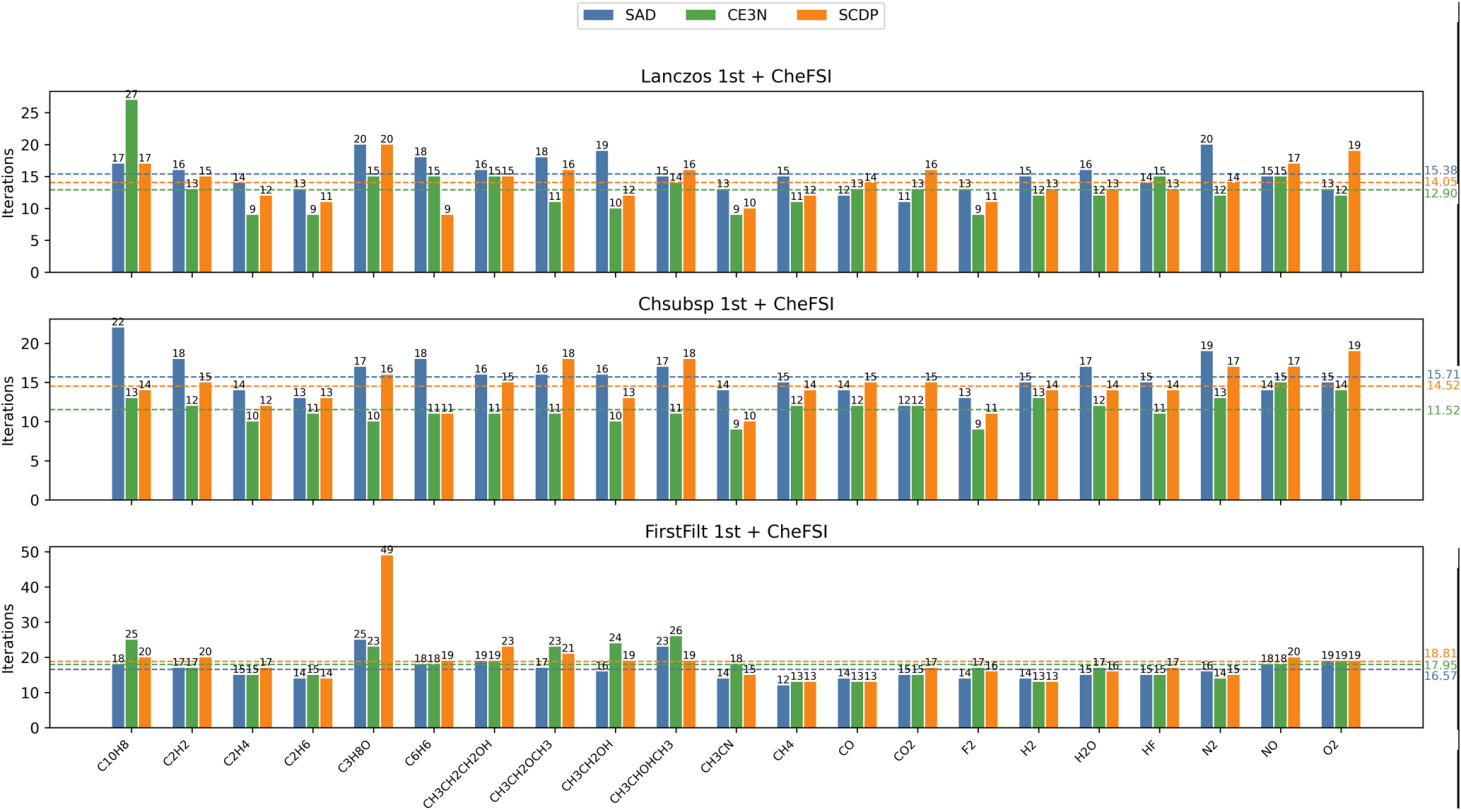
Number of SCF iterations required for convergence for the 21 selected molecules using different diagonalization strategies and initial density guesses (dashed lines indicate average values).

We first present the number of SCF iterations required for convergence in Figure 5 for different first‐iteration diagonalization methods, using SAD, CE3N, and SCDP densities as initial guesses.

From the results, we observe a clear reduction in the number of SCF iterations when ML‐predicted densities are employed in combination with the Lanczos and ChSubsp methods for the initial diagonalization. In contrast, no significant acceleration is observed for the FirstFilt approach. This difference can be attributed to the distinct characteristics of the underlying algorithms. For both Lanczos and ChSubsp, a relatively long LRR procedure is performed during the initial diagonalization stage. As a result, both the initial wavefunction and the spectral properties of the Hamiltonian are well approximated at the outset. When the initial density provided by the ML model is closer to the converged density, the corresponding wavefunction and Hamiltonian spectrum are also closer to their self‐consistent solutions. Consequently, fewer SCF iterations are required to achieve convergence. In contrast, FirstFilt performs only a brief Lanczos procedure, primarily to estimate rough spectral bounds, and the wavefunction is initialized randomly. Therefore, the electronic structure information encoded in the ML‐predicted density is not fully exploited. As a result, the potential benefits of the improved initial density are largely offset, yielding little or no observable acceleration in SCF convergence.

Beyond the impact of the diagonalization strategy, we also examine the performance differences between the ML density models themselves. The results indicate that the grid‐based model CE3N accelerates the SCF procedure more than the orbital‐based model SCDP. This improvement is particularly pronounced when ChSubsp is used for the initial diagonalization, yielding an average acceleration of 26.7%. This trend is consistent with the broader understanding that grid‐based models, while more computationally expensive to train, tend to provide higher accuracy in density prediction than orbital‐based approaches. The improved fidelity of the predicted density yields a better approximation to the converged electronic state, thereby enhancing the efficiency of subsequent SCF iterations.

It is worth noting that CE3N generally requires fewer SCF iterations than SAD when using the Lanczos and ChSubsp methods, whereas SCDP often requires more iterations in many cases. This discrepancy may be associated with differences in the underlying DFT frameworks, particularly between basis‐set‐based approaches and real‐space grid methods. CE3N is inherently formulated in real space, making it naturally compatible with grid‐based DFT implementations. In contrast, although SCDP is used to generate densities for real‐space calculations, its underlying formulation is rooted in basis‐set fitting. This mismatch may limit its ability to fully align with the real‐space representation of the Hamiltonian and electron density. As a result, CE3N demonstrates superior performance compared to SCDP, likely due to its greater alignment with the real‐space framework.

**FIGURE 6:**
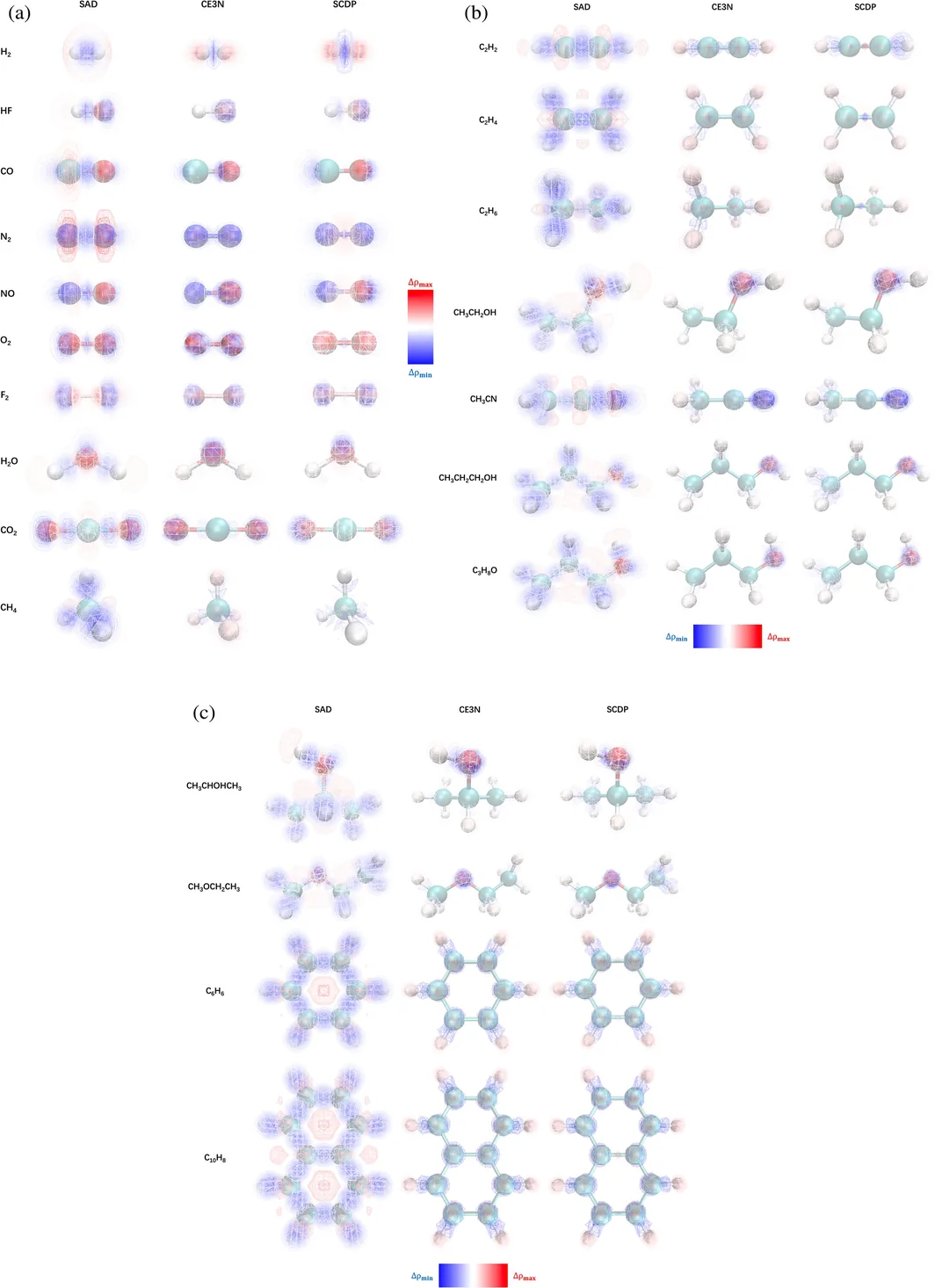
(a) Density differences between the initial guesses and the converged solutions. The molecules shown in this part are ${\text{H}}_{2},\text{HF},\text{CO},{\text{N}}_{2},\text{NO},{\text{O}}_{2},{\text{F}}_{2},{\text{H}}_{2}\text{O},{\text{CO}}_{2}$, and ${\text{CH}}_{4}$. (b) Density differences between the initial guesses and the converged solutions (continued). The molecules shown in this part are ${\text{C}}_{2}{\text{H}}_{2},{\text{C}}_{2}{\text{H}}_{4},{\text{C}}_{2}{\text{H}}_{6},{\text{CH}}_{3}{\text{CH}}_{2}\text{OH},{\text{CH}}_{3}\text{CN},{\text{CH}}_{3}{\text{CH}}_{2}{\text{CH}}_{2}\text{OH}$, and ${\text{C}}_{3}{\text{H}}_{8}\text{O}$. (c) Density differences between the initial guesses and the converged solutions (continued). The molecules shown in this part are ${\text{CH}}_{3}\text{CHOH}{\text{CH}}_{3},{\text{CH}}_{3}{\text{CH}}_{2}{\text{OCH}}_{3},{\text{C}}_{6}{\text{H}}_{6}$, and ${\text{C}}_{10}{\text{H}}_{8}$.

The intrinsic electronic structure influences the extent to which ML‐predicted densities accelerate SCF convergence. When the true molecular density closely matches the SAD initial guess, the ML density provides little additional benefit, since the starting point is already close to the converged solution. In contrast, for systems with strong bonding or pronounced electronic nonlocality, the SAD approximation may deviate significantly from the self‐consistent density. In such cases, an ML density that better captures charge redistribution can substantially improve the initial guess and reduce the number of SCF iterations. The nitrogen molecule (Figure 6a–c) illustrates this effect: its strong triple bond concentrates electron density between the nuclei, whereas SAD overlocalizes density at atomic centers. Similar trends are observed for other molecules and are further clarified by comparing the density differences between initial and converged states for both SAD and ML approaches.

We further observe that the combination of CE3N and ChSubsp tends to yield greater reductions in SCF iterations for larger systems. This trend is physically intuitive: as the number of atoms increases, the deviation of the converged electron density from the SAD initial guess generally becomes more pronounced, leaving more room for improvement with a more accurate ML‐predicted density. Consequently, larger systems may benefit more substantially from ML‐assisted initialization, thereby enhancing SCF acceleration. Although this observation warrants systematic benchmarking across broader chemical spaces and system sizes, the results suggest that ML‐assisted calculation frameworks represent a promising direction for next‐generation quantum chemistry simulations. In particular, as electronic‐structure calculations move toward exascale computing, reducing SCF iterations and other iterative computations through accurate, data‐driven initial guesses may become an increasingly important strategy to improve overall computational efficiency and scalability.

#### 3.3.2 The Density Comparison

In addition to accelerating SCF convergence using ML densities, we compare the initial and converged densities for different initial‐guess strategies to assess their quality as starting points for SCF calculations. The convergence differences for the same molecule are similar across different diagonalization strategies, indicating that the deviations between the initial and converged densities are comparable for a given initial guess. Therefore, we report only the results obtained with the Chsubsp approach.

We visualize the density deviations of the initial guess from the converged solution, $\Delta \rho =\rho_{\text{init}}-\rho_{\text{conv}}$, using isosurfaces. Regions with Δρ>0 are colored red, and those with Δρ<0 are blue, with the magnitude of the deviation indicated by the color intensity. The color scale is defined by the minimum and maximum Δρ values for each molecule. Densities are reported in units of $e^{-}\cdot {\text{Bohr}}^{-3}$. The color scale uses a spacing of 0.01 within the range [−0.1,0.1] and 0.1 outside this interval to reflect the distribution of Δρ, where most grid points have small |Δρ|, and only a few exhibit larger deviations. The corresponding results for selected molecules with different initial guesses are shown in Figure 6a–c.

From these figures, several characteristics of the three initial‐guess methods can be identified. In general, the ML densities (both CE3N and SCDP) are closer to the converged densities than SAD, particularly for larger molecules, as indicated by the smaller volume of the isosurfaces. As expected, SAD systematically underestimates the electron density in bonding regions and overestimates it around atomic centers due to the nonlocal nature of electron redistribution upon molecule formation. In contrast, CE3N and SCDP better capture the bonding regions. However, SCDP occasionally produces inaccurate predictions at the centers of certain bonds, such as in ${\text{N}}_{2}$, ${\text{O}}_{2}$, ${\text{C}}_{2}{\text{H}}_{2}$, ${\text{C}}_{2}{\text{H}}_{4}$, ${\text{C}}_{2}{\text{H}}_{6}$, ${\text{C}}_{6}{\text{H}}_{6}$, and ${\text{C}}_{10}{\text{H}}_{8}$, where a diamond‐shaped feature can be observed. This artifact likely arises from the artificial use of the O basis set to represent the bond.

Focusing on electron densities near atomic centers, both CE3N and SCDP still exhibit a localized prediction pattern, with densities at atomic cores overestimated and those in surrounding regions underestimated. This behavior is particularly noticeable for more electronegative atoms such as N, O, and F, where the outer blue isosurfaces may partially cover the inner red ones. On the contrary, the density predictions are generally more accurate for C and H atoms (except the ${\text{H}}_{2}$ molecule) and for larger organic molecules compared to small inorganic systems. This trend likely reflects the composition of the training dataset, which is dominated by organic molecules containing large numbers of C and H atoms.

To quantitatively assess the difference between each initial density and the corresponding converged SCF density, we calculated two complementary error metrics,

(23) $$D_{1}=\frac{h^{3}}{N_{e}}\underset{g}{\sum }\left|\rho_{\text{init}}(r_{g})-\rho_{\text{conv}}(r_{g})\right|,$$

and

(24) $$D_{2}={\left[\frac{\sum_{g}{\left|\rho_{\text{init}}(r_{g})-\rho_{\text{conv}}(r_{g})\right|}^{2}}{\sum_{g}{\left|\rho_{\text{conv}}(r_{g})\right|}^{2}}\right]}^{1/2},$$

where h is the real‐space grid spacing and $N_{e}$ is the number of electrons. The dimensionless quantity $D_{1}$ is the integrated absolute density difference normalized per electron and therefore measures the overall amount of density that must be redistributed to transform the initial density into the converged density. Because it weights all grid‐point deviations linearly, it primarily reflects the global density mismatch and permits a meaningful comparison among molecules of different sizes. In contrast, $D_{2}$ is a relative $L^{2}$ error normalized by the $L^{2}$ norm of the converged density. Its quadratic weighting gives greater importance to large, spatially localized deviations, making it more sensitive to errors near atomic centers, chemical bonds, and other regions of high density.

**FIGURE 7:**
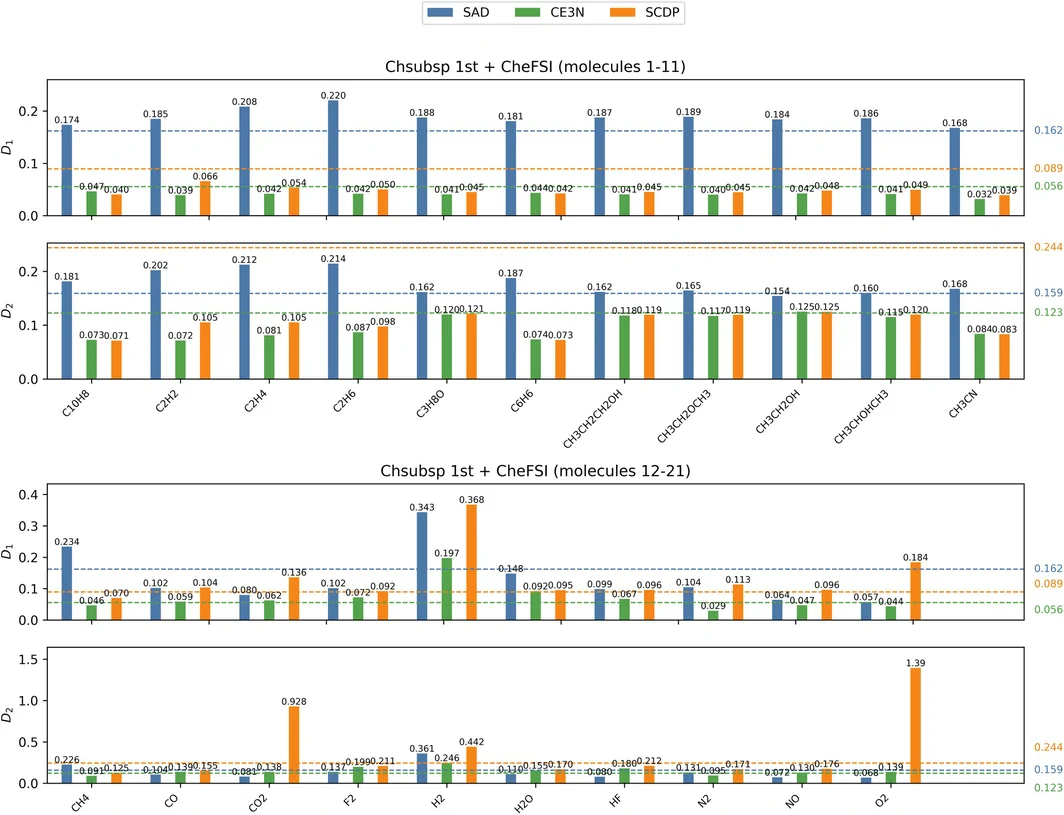
Quantitative comparison of the initial and converged electron densities for the 21 selected molecules using different initial density guesses. The upper panel shows the integrated absolute density difference per electron, $D_{1}$. The lower panel shows the relative $L^{2}$ density error, $D_{2}$. (dashed lines denote the average values)

The results in Figure 7 show that CE3N provides the smallest average errors for both metrics. Its mean $D_{1}$ is 0.0555, compared with 0.1620 for SAD and 0.0894 for SCDP. CE3N also gives the smallest $D_{1}$ for 19 of the 21 molecules, with SCDP being only marginally smaller for benzene and naphthalene. This demonstrates that CE3N provides the most consistently accurate global distribution of the initial electron density. For $D_{2}$, the mean values are 0.1226, 0.1591, and 0.2439 for CE3N, SAD, and SCDP, respectively. The molecule‐resolved $D_{2}$ results are more varied, however: CE3N gives the lowest value for 10 molecules, SAD for 7, and SCDP for 4. In particular, SAD retains smaller $D_{2}$ values for several small heteronuclear, oxygen‐containing, or multiple‐bonded systems, including CO, ${\text{CO}}_{2}$, ${\text{F}}_{2}$, ${\text{H}}_{2}\text{O}$, HF, NO, and ${\text{O}}_{2}$. This indicates that, although CE3N substantially reduces the integrated density error in these systems, some localized deviations may remain. The large SCDP $D_{2}$ values for ${\text{CO}}_{2}$ and ${\text{O}}_{2}$, approximately 0.928 and 1.394, respectively, further reveal strongly localized density errors that are less apparent from the integrated $D_{1}$ metric. Taken together, the two metrics show that CE3N gives the most robust overall initial‐density improvement across the test set, while $D_{2}$ and the density‐difference isosurfaces provide complementary information about the location and severity of residual localized errors.

#### 3.3.3 The One‐Shot Calculation Based on the Initial Density

**FIGURE 8:**
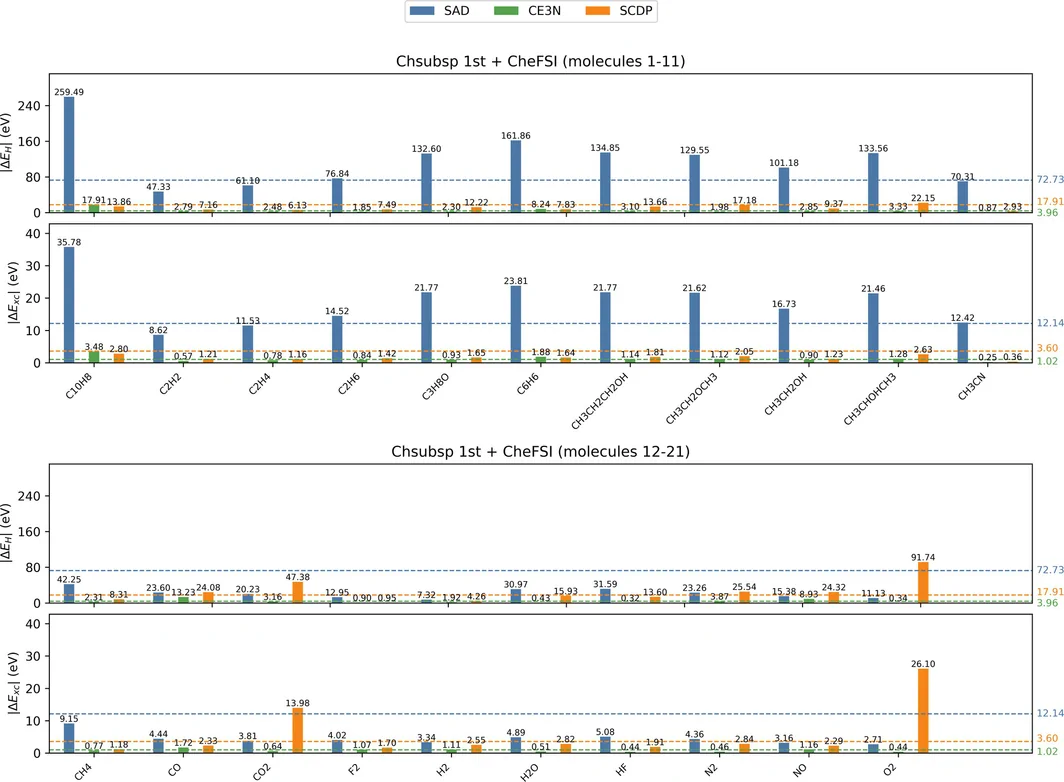
The absolute difference between the one‐shot Hartree and exchange‐correlation potentials computed using the ML densities and the converged ones.

Next, for different initial‐density strategies, we examine the feasibility of performing one‐shot non‐self‐consistent (NSC) calculations and directly comparing the results with fully converged solutions as a reference. Figure 8 shows the absolute differences between the initial one‐shot and converged Hartree and exchange‐correlation energies, denoted as $\left|\Delta E_{H}\right|$ and $\left|\Delta E_{xc}\right|$, respectively. As mentioned earlier, the differences in the converged densities across diagonalization strategies are similar for a given initial density guess. Consequently, the corresponding Hartree and exchange‐correlation potential energies obtained from the same initial density are also nearly identical across these strategies. Therefore, for clarity, we present only the results from the ChSubsp strategy, as they are nearly identical to those from other diagonalization methods.

CE3N yields the most robust density predictions, as indicated by the lowest average values of $\left|\Delta E_{H}\right|$ and $\left|\Delta E_{xc}\right|$ compared to SCDP. As expected, the SAD results deviate substantially from the converged values, particularly for larger systems. The behavior of SCDP merits attention. In certain cases, SCDP yields even larger $\left|\Delta E_{H}\right|$ and $\left|\Delta E_{xc}\right|$ than SAD, especially for small oxygen‐containing molecules. Notable deviations are observed for $CO_{2}$ and $O_{2}$, with smaller but still discernible discrepancies for CO and NO. This behavior may be associated with the basis‐set construction underlying the SCDP model, which employs oxygen atomic orbitals to fit the density in bonding regions [64]. Such a representation may introduce additional fitting errors in systems where oxygen plays a dominant role in bond formation. More broadly, the previously discussed better alignment of grid‐based approaches with real‐space KS‐DFT relative to orbital‐based methods likely also contributes to CE3N's superior overall performance.

The magnitudes of $\left|\Delta E_{H}\right|$ and $\left|\Delta E_{xc}\right|$ remain significant when compared to the conventional standard of chemical accuracy (0.01 eV/atom). This is not unexpected, as errors in the predicted density propagate directly into the evaluation of the corresponding potential energy contributions. At present, the accuracy may not yet be sufficient to rely on ML‐predicted densities alone for quantitatively precise predictions of density‐dependent physical properties. Nevertheless, these results provide an important intermediate validation of the density predictions and highlight the promise of this approach. Continued improvements in ML‐based density modeling may ultimately enable a more direct realization of the fundamental ground‐state DFT principle that knowing the exact electron density determines all ground‐state properties of the system [1].

#### 3.3.4 The Energy Comparison

**FIGURE 9:**
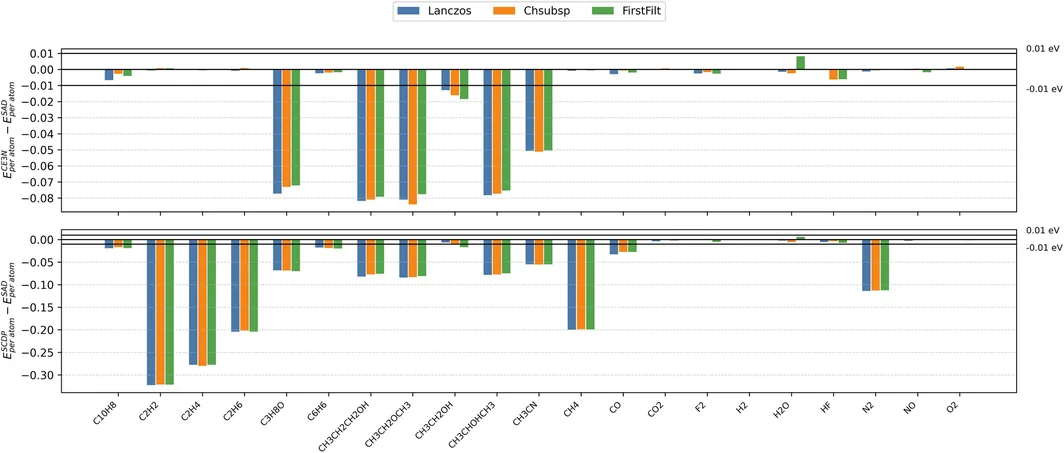
The energy per atom differences between the ML densities and SAD.

Finally, we compare the per‐atom energy differences between ML‐predicted densities and the SAD initial guess. The results for the different methods are presented in Figure 9.

The results indicate that, for most molecules, CE3N yields per‐atom energies nearly identical to those obtained with SAD. Notably, for ethanol, $CH_{3}\mathrm{CN}$, and various isomers of $C_{3}H_{8}O$, CE3N even produces lower energies. SCDP exhibits a similar overall trend and, in addition, yields much lower energies for the nitrogen molecule, methane, ethane, ethene, and ethyne.

One possible explanation is the presence of multiple local minima in the KS‐DFT energy landscape within the accessible wavefunction space. An SCF procedure initialized from the SAD density may become trapped in one of these higher‐energy minima. In contrast, an ML‐predicted density may help the SCF iterations escape such traps and converge to a lower‐energy minimum, potentially even the global minimum.

However, the energy lowering observed for the small organic molecules mentioned above is unusually large. For example, ethyne exhibits an energy reduction of about 1.2 eV, which is significant for such a small molecule. From the density comparison, one can observe a noticeable increase in the converged density relative to the SCDP initial guess at the center of the bond in the $N_{2}$ molecule (Figure 6a). Similar pronounced increases or decreases in the bond‐center density are also observed for $C_{2}H_{2}$, $C_{2}H_{4}$, and $C_{2}H_{6}$ (Figure 6b). Given the characteristics of the SCDP density predictions, this large energy lowering may arise from an artificial charge density appearing at the bond centers. In the SCDP model, the density in bonding regions is represented using the basis functions of an O atom, which can introduce artifacts. Although, this representation performs well for larger organic molecules, it may behave poorly for smaller systems, particularly in bonding regions. As a result, the initial density may place the SCF procedure in a nonphysical region of the energy landscape, leading to convergence toward an artificial minimum.

### 3.4 GPU Acceleration With CuPy

**FIGURE 10:**
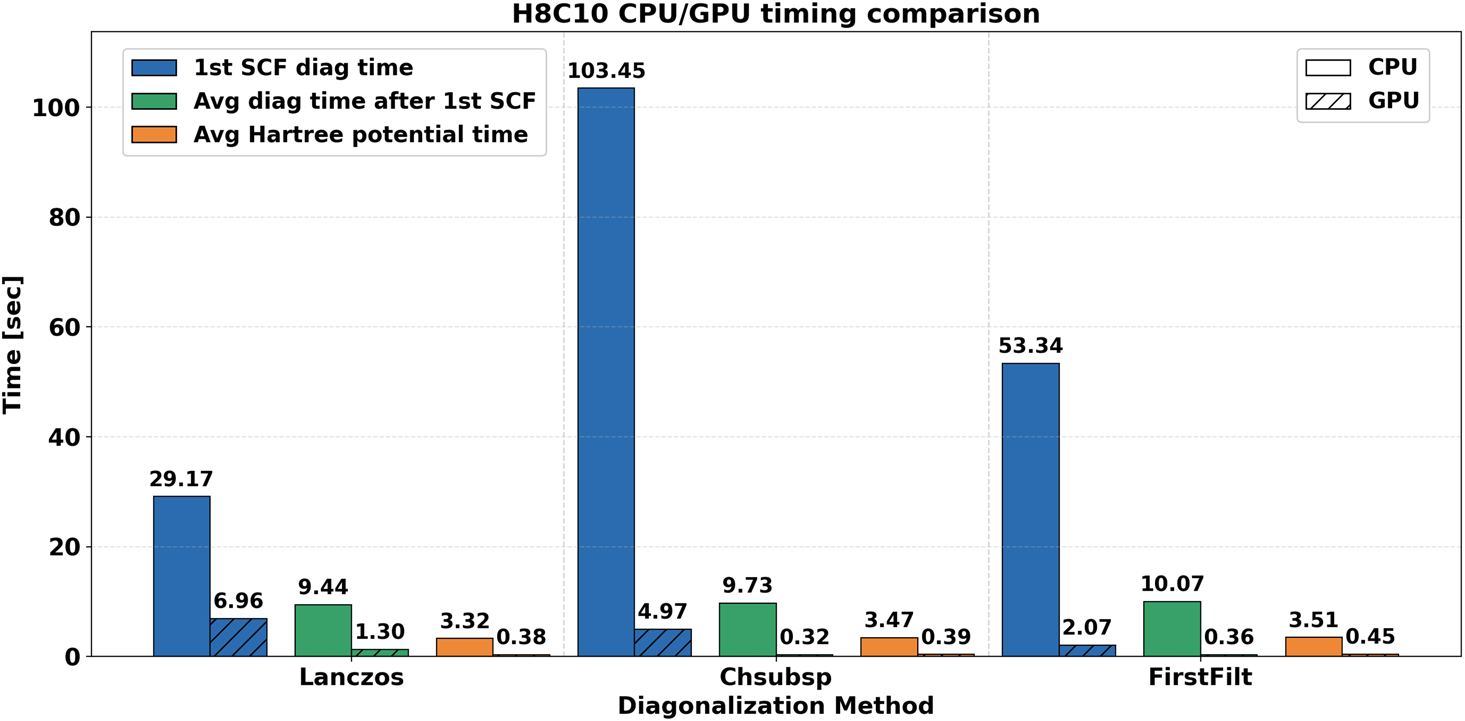
CPU/GPU timing comparison for ${\text{C}}_{10}{\text{H}}_{8}$ using different first‐SCF diagonalization strategies. The Figure shows the diagonalization time for the first SCF iteration, the average diagonalization time after the first SCF iteration, and the average Hartree‐potential update time. Calculations were performed on an AMD Ryzen 9 8945X CPU and an NVIDIA GeForce RTX 5070 GPU.

In addition to reducing the number of SCF iterations through improved initial density guesses, the overall wall time can also be lowered by accelerating the computational kernels within each SCF step. To examine this aspect, we implemented a GPU backend in PARSEC.py using CuPy. We compared CPU and GPU timings for ${\text{C}}_{10}{\text{H}}_{8}$, reporting the diagonalization time in the first SCF iteration, the average diagonalization time after the first SCF iteration, and the average Hartree‐potential update time. The calculations were performed using an AMD Ryzen 9 8945X CPU and an NVIDIA GeForce RTX 5070 GPU. The results are summarized in Figure 10.

GPU execution substantially accelerates both the first‐step diagonalization and the subsequent SCF iterations. The first SCF diagonalization is accelerated by approximately 4.2×, 20.8×, and 25.8× for Lanczos, ChSubsp, and FirstFilt, respectively, while the average diagonalization time after the first SCF iteration is reduced by about 7.3×, 30.4×, and 28.0×. The Hartree‐potential update is also accelerated by roughly 8× across all cases. The particularly large gains observed for ChSubsp and FirstFilt suggest that repeated Hamiltonian applications and filtering operations benefit strongly from accelerator execution. Overall, these results demonstrate that CuPy‐based GPU acceleration effectively reduces the cost of the dominant numerical kernels in PARSEC.py and complements ML‐assisted initialization by reducing the computational cost per SCF iteration.

We emphasize that the present CPU/GPU timing comparison reflects the current PARSEC.py implementation rather than a fully optimized multicore CPU‐versus‐GPU benchmark. In earlier versions of Python and in the usual GIL‐enabled CPython build, CPU‐bound Python threads do not achieve true parallel execution. Free‐threaded Python was introduced in Python 3.13 and is supported in Python 3.14, the latest stable major release; however, it remains optional. Since MPI‐based CPU parallelization has not yet been implemented in PARSEC.py, the CPU timings are best viewed as a practical baseline for the current workflow. From this perspective, Figure 10 highlights the immediate benefit of GPU acceleration, while further CPU‐side improvements through MPI parallelization remain a direction for future work.

## 4 Conclusions

In this work, we present PARSEC.py, a Python‐based real‐space KS‐DFT framework that provides a modern, transparent, and extensible platform for first‐principles electronic‐structure calculations. By discretizing the KS equations on uniform real‐space grids, the framework avoids basis‐set approximations while naturally yielding sparse Hamiltonian matrices well suited for large‐scale parallel computation. The implementation leverages the Python scientific ecosystem, allowing seamless integration with high‐performance numerical libraries and ML frameworks while maintaining code clarity and modularity.

We described the theoretical foundations and algorithmic components of the framework, including the real‐space representation of the Hamiltonian, FD discretization of the Laplacian, pseudopotential treatment, and efficient diagonalization strategies based on Lanczos procedures, Chebyshev filtering, and RR steps. These algorithms enable scalable solutions to the large eigenvalue problems inherent in real‐space DFT. In addition, we detailed the implementation of the SCF procedure, including occupation and density updates, Poisson equation solutions for the Hartree potential, and stabilization through mixing schemes.

A central feature of PARSEC.py is its capability to incorporate ML charge densities as initial guesses for SCF iterations. In contrast to the conventional SAD method, ML densities provide a closer starting point that can significantly accelerate convergence. This framework establishes a flexible interface between modern AI models and traditional electronic‐structure methods. Calculations on representative molecular systems demonstrate that, with an appropriate choice of diagonalization method, ML‐initialized densities can reduce the number of SCF iterations and improve overall computational efficiency while maintaining the accuracy expected from KS‐DFT calculations. These results highlight the potential of ML‐assisted initialization strategies to accelerate electronic‐structure calculations, particularly for large systems, where SCF convergence is often the dominant computational cost.

In addition to ML‐assisted SCF acceleration, PARSEC.py leverages CuPy‐based GPU acceleration for key kernels, including Hamiltonian diagonalization and Hartree‐potential updates, thereby reducing the cost of the dominant steps in the present workflow. Along with continued advances in GPU hardware and software, these results highlight the promise of real‐space DFT for large‐scale and exascale‐oriented computing. Future work will focus on MPI‐based CPU parallelization and on extending the GPU implementation to cluster environments to improve scalability.

Overall, PARSEC.py provides a user‐ and developer‐friendly real‐space KS‐DFT platform that combines algorithmic rigor with modern software design. Its Python‐based architecture facilitates rapid method development, algorithmic experimentation, integration with emerging AI tools, and GPU parallelization. Looking forward, the framework opens opportunities for further developments, including advanced exchange‐correlation functionals, diversification of PPs, improved parallelization strategies, and deeper integration with ML models for predicting electronic quantities. Such efforts will help bridge the gap between data‐driven approaches and traditional electronic‐structure theory, enabling more efficient, scalable simulations of complex chemical and materials systems.

## Author Contributions

J.Q. acquired funding for, and led the project. Z.Z. wrote the code and first manuscript under the supervision of J.Q. C.M.P. streamlined ML models to predict the initial charge densities for targeted molecules. P.K. contributed to the development of the GPU acceleration code. All authors contributed to reviewing and editing the manuscript.

## Funding

This work was supported by the Early Career Research Program of the U.S. Department of Energy, Office of Science, Office of Basic Energy Sciences, Chemical Sciences, Geosciences, and Biosciences Division, through Contract No. DE‐AC02‐05CH11231. This research used resources of the National Energy Research Scientific Computing Center, a DOE Office of Science User Facility supported by the Office of Science of the U.S. Department of Energy under Contract No. DE‐AC02‐05CH11231 with NERSC award BES‐ERCAP0036640.

## Conflicts of Interest

The authors declare no conflicts of interest.

## Supporting information




**Section S1:** Lanczos procedure.
**Section S2:** Orthonormalization methods.
**Table S1:** Default parameters, iteration pattern, and usage for the diagonalization methods.
**Section S4:** Update occupation numbers by the bisection procedure.
**Figure S1:** The bisection procedure for updating occupation numbers and the Fermi level.
**Figure S2:** The CG method for updating Hartree potential through Poisson equation.
**Table S2:** Box‐size convergence test for ${\text{C}}_{10}{\text{H}}_{8}$ with a fixed grid spacing of 0.2Å.
**Table S3:** Validation of PARSEC.py against the original PARSEC code for the 21 molecules considered in this work.

## Data Availability

The data that support the findings of this study are available on request from the corresponding author. The source code for the calculations is openly available on the github at https://github.com/QianGroupPage/PARSEC.py.
