# Supplementary material for: PARSEC.py: A Python‐Based Real‐Space Kohn–Sham Density Functional Theory Code Accelerated by Machine Learned Charge Density
Source: J Comput Chem. 2026 Aug 21;47(23):e70482. doi: 10.1002/jcc.70482 (PMC13496248; doi:10.1002/jcc.70482)
Supplement: Supplementary file 1 [file JCC-47-0-s001.pdf]

Supporting Information for: PARSEC.py: A Python-based Real-space Kohn-Sham Density Functional Theory Code Accelerated by Machine Learned Charge Density

Zeyi Zhang,[†,‡] Carlos Mora Perez,[‡] Patrick Kwon,[†] Martin Head-Gordon,[†,‡] and Jin Qian[*,‡]

†*Department of Chemistry, University of California, Berkeley, California 94720, USA*

‡*Chemical Sciences Division, Lawrence Berkeley National Laboratory, Berkeley CA 94720, USA.*

E-mail: jqian2@lbl.gov

1 Lanczos procedure

Let $\boldsymbol{H} \in \mathbb{C}^{n\times n}$ be a Hermitian matrix, $\vec{v}_1$ be a normalized random starting vector, and set $\vec{v}_0 = \vec{0}$, $\beta_1 = 1$, $\vec{w}_1 = \vec{v}_1$. Then the Lanczos procedure generates an orthonormal basis $\{\vec{v}_k\}$ by a three-term recurrence

$$
\begin{aligned}
&\vec{v}_{k+1} = \frac{\boldsymbol{H}\vec{v}_k - \alpha_k\vec{v}_k - \beta_k\vec{v}_{k-1}}{\beta_{k+1}} \quad\quad k = 1, 2, ..., m \\
&\alpha_k = \vec{v}_k^\dagger \boldsymbol{H}\vec{v}_k \quad\quad \vec{w}_{k+1} = \boldsymbol{H}\vec{v}_k - \alpha_k\vec{v}_k - \beta_k\vec{v}_{k-1} \quad\quad \beta_k = \|\vec{w}_k\|
\end{aligned}
\tag{1}
$$

$\vec{v}_{k+1}$ is constructed by orthogonalizing $\boldsymbol{H}\vec{v}_k$ against $\vec{v}_k$ and $\vec{v}_{k-1}$. For $k = 1$, Eq. 1 ensures $\vec{v}_2 \perp \vec{v}_1$ with $\beta_2 = \vec{v}_1^\dagger \boldsymbol{H}\vec{v}_2$ implied by Hermiticity. Then it produces an orthonormal set $\{\vec{v}_1, \vec{v}_2, \vec{v}_3\}$ when $k = 2$, given β_2 is the projection of $\boldsymbol{H}\vec{v}_2$ onto $\vec{v}_1$. After m iterations, $\vec{v}_1, \ldots, \vec{v}_m$, by mathematical induction, forms an orthonormal basis of the Krylov subspace:

$$
\mathcal{K}_m(\boldsymbol{H}, \vec{v}_1) = \mathrm{span}\{\vec{v}_1, \boldsymbol{H}\vec{v}_1, \ldots, \boldsymbol{H}^{m-1}\vec{v}_1\}. \tag{2}
$$

Defining Lanczos vectors $\boldsymbol{V}_m = [\vec{v}_1, \ldots, \vec{v}_m]$, the projection of $\boldsymbol{H}$ in the subspace is a tridiagonal matrix:

$$
\boldsymbol{T}_m = \boldsymbol{V}_m^\dagger \boldsymbol{H}\boldsymbol{V}_m = \begin{bmatrix} \alpha_1 & \beta_2 & \cdots & 0 \\ \beta_2 & \alpha_2 & \ddots & \vdots \\ \vdots & \ddots & \ddots & \beta_m \\ 0 & \cdots & \beta_m & \alpha_m \end{bmatrix} \tag{3}
$$

Diagonalizing $\boldsymbol{T}_m$ gives eigenvalues $\boldsymbol{\Lambda}_m = \mathbf{diag}(\lambda_1, \ldots, \lambda_m)$ and corresponding eigenvectors $\boldsymbol{Q}_m = [\vec{q}_1, \ldots, \vec{q}_m]$. The eigenpairs $\{\boldsymbol{V}_m\boldsymbol{Q}_m, \boldsymbol{\Lambda}_m\}$ approximate those of $\boldsymbol{H}$, with rapid convergence for extremal eigenvalues.

$$
\boldsymbol{T}_m\boldsymbol{Q}_m = \boldsymbol{\Lambda}_m\boldsymbol{Q}_m = (\boldsymbol{V}_m^\dagger \boldsymbol{H}\boldsymbol{V}_m)\boldsymbol{Q}_m \Rightarrow \boldsymbol{H}(\boldsymbol{V}_m\boldsymbol{Q}_m) = \boldsymbol{\Lambda}_m(\boldsymbol{V}_m\boldsymbol{Q}_m) \tag{4}
$$

2 Orthonormalization methods

Let $\boldsymbol{A} = [\vec{a}_1, \vec{a}_2, ..., \vec{a}_m]$ denote the original basis and $\boldsymbol{Q} = [\vec{q}_1, \vec{q}_2, ..., \vec{q}_m]$ the orthonormalized basis.

For the Gram–Schmidt process,[1] starting with $\vec{q}_1 = \vec{a}_1/\|\vec{a}_1\|$, the orthonormal basis is constructed recursively by removing components along previously computed vectors:

$$\vec{q}_k = \frac{(\boldsymbol{I} - \sum_{i=1}^{k-1} \vec{q}_i\vec{q}_i^{\dagger})\vec{a}_k}{\|(\boldsymbol{I} - \sum_{i=1}^{k-1} \vec{q}_i\vec{q}_i^{\dagger})\vec{a}_k\|} \quad k = 2, 3, ..., m \tag{5}$$

The Cholesky–QR algorithm is carried out as follows.[2]

$$\boldsymbol{G} = \boldsymbol{A}^{\dagger}\boldsymbol{A} \rightarrow \boldsymbol{G} = \boldsymbol{R}^{\dagger}\boldsymbol{R} \xrightarrow[\text{triangular } R]{\text{solve upper}} \boldsymbol{Q} = \boldsymbol{A}\boldsymbol{R}^{-1} \tag{6}$$

The orthonormality $\boldsymbol{Q}^{\dagger}\boldsymbol{Q} = \boldsymbol{I}$ follows directly, with $\boldsymbol{R}$ and $\boldsymbol{R}^{-1}$ computed from element-wise relations.

For Householder QR,[3] with $\vec{e}_1 = [1, 0, ..., 0]^{\mathsf{T}}$, we construct a transformation that zeros all but the first component of $\vec{a}_1$:

$$\boldsymbol{Q}_1\vec{a}_1 = \alpha_1\vec{e}_1 \quad \boldsymbol{Q}_1 = \boldsymbol{I} - 2\vec{v}_1\vec{v}_1^{\dagger} \quad \vec{v}_1 = \frac{\vec{u}_1}{\|\vec{u}_1\|} \quad \vec{u}_1 = \vec{a}_1 - \alpha_1\vec{e}_1 \quad \alpha_1 = \|\vec{a}_1\| \tag{7}$$

$\boldsymbol{Q}_1\boldsymbol{A}$ also modifies $\vec{a}_2, ..., \vec{a}_m$. Removing its first row and column yields the submatrix $\boldsymbol{A}' = [\vec{a}'_2, ..., \vec{a}'_m]$, from which a second transformation $\boldsymbol{Q}'_2$, constructed as in Eq. 7, eliminates all but the first entry of $\vec{a}'_2$. Repeating this process recursively transforms $\boldsymbol{A}$ to an upper-triangular matrix $\boldsymbol{R}$ and yields the orthonormal basis $\boldsymbol{Q}$:

$$\boldsymbol{Q}_1\boldsymbol{A} = \begin{bmatrix} \alpha_1 & * & \cdots & * \\ 0 & & & \\ \vdots & & \boldsymbol{A}' & \\ 0 & & & \end{bmatrix} \xrightarrow[\boldsymbol{Q}_2 = \begin{bmatrix} 1 & 0 \\ 0 & \boldsymbol{Q}'_2 \end{bmatrix}]{\text{from } \boldsymbol{A}' \text{ build}} \cdots \xRightarrow[\text{iterations}]{m-1} \begin{cases} \boldsymbol{R} = \boldsymbol{Q}_{m-1}\cdots\boldsymbol{Q}_1\boldsymbol{A} \\ \boldsymbol{Q} = \boldsymbol{Q}_1\cdots\boldsymbol{Q}_{m-1} \end{cases} \tag{8}$$

$\boldsymbol{Q}^{\dagger}\boldsymbol{Q} = \boldsymbol{I}$ and $\boldsymbol{A} = \boldsymbol{Q}\boldsymbol{R}$ follow from the unitarity of $\boldsymbol{Q}_1, \cdots, \boldsymbol{Q}_{m-1}$ implied by the construction in Eq. 7.

3 Diagonalization methods

The detailed diagonalization algorithms of Chsubsp, FirstFilt and CheFSI are described below and Table S1 summarizes the default parameters, iteration pattern, and usage of all diagonalization methods.

3.1 Lanczos

Lanczos performs the LRR process with a large m and its eigenpairs $\{\boldsymbol{\Psi}_{n\times n'_{ev}}, \boldsymbol{\varepsilon}_{n'_{ev}}\}$ approximate those of $\boldsymbol{H}$.

3.2 Chsubsp

ChSupsb begins with an LRR process using a large m to obtain the initial eigenpairs $\{\boldsymbol{\Phi}^{(0)}_{n\times n'_{ev}} = \boldsymbol{\Psi}_{n\times n'_{ev}}, \boldsymbol{\varepsilon}^{(0)}_{n'_{ev}} = \boldsymbol{\varepsilon}_{n'_{ev}}\}$, and spectral bounds.[4] The upper bound is estimated as $b = \max_k\{|\lambda_k|\} + \beta_m$,[5,6] where λ_k ($k \in \{1, \ldots, m\}$) and β_m are drawn from $\boldsymbol{\Lambda}_m$ and $\boldsymbol{T}_{m\times m}$, the lower bound and the scaling parameter are set to $a^{(0)} = \epsilon_{n'_{ev}}$ and $a_0^{(0)} = \epsilon_1$, with $\epsilon_{n'_{ev}}$ and ϵ_1 from $\boldsymbol{\varepsilon}_{n'_{ev}}$. A CRR loop with normalized Chebyshev filtering and $N_{\max} = 30$ is then performed. If convergence is achieved after $N_c(\leq N_{\max})$ iterations or the maximum iteration is reached, the resulting eigenpairs $\{\boldsymbol{\Phi}^{(N_c)}_{n\times n'_{ev}}, \boldsymbol{\varepsilon}^{(N_c)}_{n'_{ev}}\}$ are taken as approximations to those of $\boldsymbol{H}$.

3.3 FirstFilt

FirstFilt starts with a special short Lanczos procedure ($m = 6\sim10$) to estimate initial upper and lower bounds.[7,8] In the kth iteration ($2 \leq k \leq m$) of the special Lanczos recurrence, if $\beta_k < 0.01$, it is replaced by $10\beta_k$ in the subsequent step to obtain more conservative upper and lower bounds, and the procedure terminates early if $\beta_k < 2.5 \times 10^{-16}$ even after this scaling. Let q_{lk} denote the lth component of the kth eigenvector in $\boldsymbol{Q}_{m\times m}$. Three upper bounds are formed: $b_1 = \lambda_m + \beta_m$, $b_2 = \lambda_m + |q_{mm}| \cdot \beta_m$, and $b_3 = \lambda_m + \max_k\{|q_{mk}|\} \cdot \beta_m$, and combined as the final upper bound $b = b_1/4 + b_2/4 + b_3/2$. The inital lower bound is calculated as $a^{(0)} = 0.8\lambda_1 + 0.2\lambda_{m-1}$. The initial eigenvectors $\boldsymbol{\Phi}^{(0)}_{n\times n'_{ev}}$ are randomly initialized, followed by a CRR loop with direct Chebyshev filtering and $N_{\max} = 3\sim5$, depending on the polynomial degree M. Upon the convergence after $N_f(\leq N_{\max})$ iterations or the maximum iteration is reached, the eigenpairs $\{\boldsymbol{\Phi}^{(N_f)}_{n\times n'_{ev}}, \boldsymbol{\varepsilon}^{(N_f)}_{n'_{ev}}\}$ approximate those of $\boldsymbol{H}$.

3.4 CheFSI

CheFSI is applied only after the first SCF iteration.[5] We denote the SCF iteration index by the left superscript $[j]$. Then at the jth SCF iteration, it initializes the eigenpairs from the previous SCF step,

$\{{}^{[j]}\boldsymbol{\Phi}^{(0)}_{n\times n'_{ev}} = {}^{[j-1]}\boldsymbol{\Phi}_{n\times n'_{ev}}, {}^{[j]}\boldsymbol{\varepsilon}^{(0)}_{n'_{ev}} = {}^{[j-1]}\boldsymbol{\varepsilon}_{n'_{ev}}\}$, and estimates spectral bounds via a short Lanczos procedure ($m = 6$). As in the ChSupsb approach, the bounds and scaling parameter are set to $b = \max_k |\lambda_k| + \beta_m$, $a^{(0)} = {}^{[j-1]}\epsilon_{n'_{ev}}$, and $a_0^{(0)} = {}^{[j-1]}\epsilon_1$. A single CRR step with normalized Chebyshev filtering is then performed, yielding updated eigenpairs $\{{}^{[j]}\boldsymbol{\Phi}_{n\times n'_{ev}} = {}^{[j]}\boldsymbol{\Phi}^{(1)}_{n\times n'_{ev}}, {}^{[j]}\boldsymbol{\varepsilon}_{n'_{ev}} = {}^{[j]}\boldsymbol{\varepsilon}^{(1)}_{n'_{ev}}\}$ to approximate those of $\boldsymbol{H}$.

Table S1: Default parameters, iteration pattern, and usage for the diagonalization methods

**Method**	Lanczos	ChSubsp	FirstFilt	CheFSI
**Number of requested eigenpairs n_{ev}**	$\max\{16, \lceil 0.7N_e + 0.5\rceil\}$, $\lceil\cdot\rceil$ is the ceiling function			
**Number of extracted eigenpairs n'_{ev}**	$n_{ev} + 15$	$n_{ev} + 15$	$n_{ev} + 15$	n_{ev}
**Truncated size m_0**	$\min\{(\min\{1.8^{m/m'}, 3.2\})n'_{ev}, m\}$,<br>$m'$ is the number of nondegenerate eigenvalues with a degeneracy tolerance of 10^{-5}			
**Lanczos procedure or LRR step m**	LRR<br>$n'_{ev} + 500$	LRR<br>$\max\{3n'_{ev}, 450\}$	Lanczos procedure<br>6	Lanczos procedure<br>6
**Initial wavefunction $\boldsymbol{\Phi}^{(0)}_{n\times n'_{ev}}$**	N/A	$\boldsymbol{H}_{n\times n}$<br>$\Downarrow$ LRR<br>$\boldsymbol{\Psi}_{n\times n'_{ev}}$	randomly initialized	$\boldsymbol{\Phi}_{n\times n'_{ev}}$<br>from previous SCF iteration
**Initial bounds $\{a_0^{(0)}, a^{(0)}, b\}$**	N/A	$\boldsymbol{H}_{n\times n}$<br>$\downarrow$ LRR<br>$\{\boldsymbol{T}_{m\times m}, \boldsymbol{\Lambda}_m\}, \boldsymbol{\varepsilon}_{n'_{ev}}$<br>$\Downarrow$<br>$a_0^{(0)} = \epsilon_1, a^{(0)} = \epsilon_{n'_{ev}}$<br>$b = \max_k\{\|\lambda_k\|\} + \beta_m$	$\boldsymbol{H}_{n\times n}$<br>Lanczos $\downarrow$ procedure<br>$\{\boldsymbol{T}_{m\times m}, \boldsymbol{Q}_{m\times m}, \boldsymbol{\Lambda}_m\}$<br>$\Downarrow$<br>$a^{(0)} = 0.8\lambda_1 + 0.2\lambda_{m-1}$<br>$b = \frac{1}{4}b_1 + \frac{1}{4}b_2 + \frac{1}{2}b_3$<br>$\begin{cases} b_1 = \lambda_m + \beta_m \\ b_2 = \lambda_m + \|q_{mm}\| \cdot \beta_m \\ b_3 = \lambda_m + \max_k\{\|q_{mk}\|\} \cdot \beta_m \end{cases}$	$\boldsymbol{H}_{n\times n}$<br>Lanczos $\downarrow$ procedure<br>$\{\boldsymbol{T}_{m\times m}, \boldsymbol{\Lambda}_m\}$<br>+<br>$\boldsymbol{\varepsilon}_{n'_{ev}}$<br>from previous SCF iteration<br>$\Downarrow$<br>$a_0^{(0)} = \epsilon_1, a^{(0)} = \epsilon_{n'_{ev}}$<br>$b = \max_k\{\|\lambda_k\|\} + \beta_m$
**Filter type**	N/A	normalized Chebyshev	Chebyshev	normalized Chebyshev
**CRR loop settings $\{M, N_{\max}, e_{\text{tol}}\}$**	N/A	$\{10, 30, 0.05\}$	$\{10, 5, 0.08\}$	$\{10, 1, \text{N/A}\}$
**Usage**	first SCF	first SCF	first SCF	after first SCF

The notations are consistent with those used in the main comntext.

4 Update occupation numbers by the bisection procedure

After diagonalizing the Hamiltonian $\boldsymbol{H}$, we obtain the eigenpairs $\{\boldsymbol{\Phi}_{n\times n_{ev}}, \boldsymbol{\varepsilon}_{n_{ev}}\}$. Since the Fermi level ϵ_F is unknown, the occupation numbers $\{f_i(\epsilon_F)\}$ are computed from Fermi-Dirac statistics

$$f_i(\epsilon_F) = \frac{1}{1+e^{(\epsilon_i-\epsilon_F)/kT}} \qquad 2\sum_{i=1}^{n_{occ}} f_i(\epsilon_F) = n_e \tag{9}$$

using the bisection method.[9] We define

$$d_x = 2\sum_{i=1}^{n_{ev}} f_i(x) - n_e \tag{10}$$

which measures the deviation between the total occupation with a trial Fermi level x and the number of valence electrons n_e. We initialize the bracketing interval by $a = \epsilon_1 - 1$ and $b = \epsilon_{\lceil n_e/2\rceil} + 1$. Towards zero temperature, these two bounds place ϵ_F below and above all occupied states, respectively; thus, at moderate temperatures, this choice typically ensures $d_a < 0$ and $d_a > 0$. If $d_a d_b > 0$, we assign full occupation to the lowest orbitals by default. Otherwise, we perform standard bisection with $c = (a+b)/2$, updating the interval according to the sign of d_c, until $|d_c| < e_{\text{tol}}$ or the maximum iteration number $N_{\max}$ is reached. The resulting $\{f_i(c)\}$ are taken as the final occupations. The bisection procedure is summarized in Figure S1, where (k) denotes the iteration index. In the implementation, the default parameters are set to $e_{\text{tol}} = 10^{-6}$ and $N_{\max} = 200$.

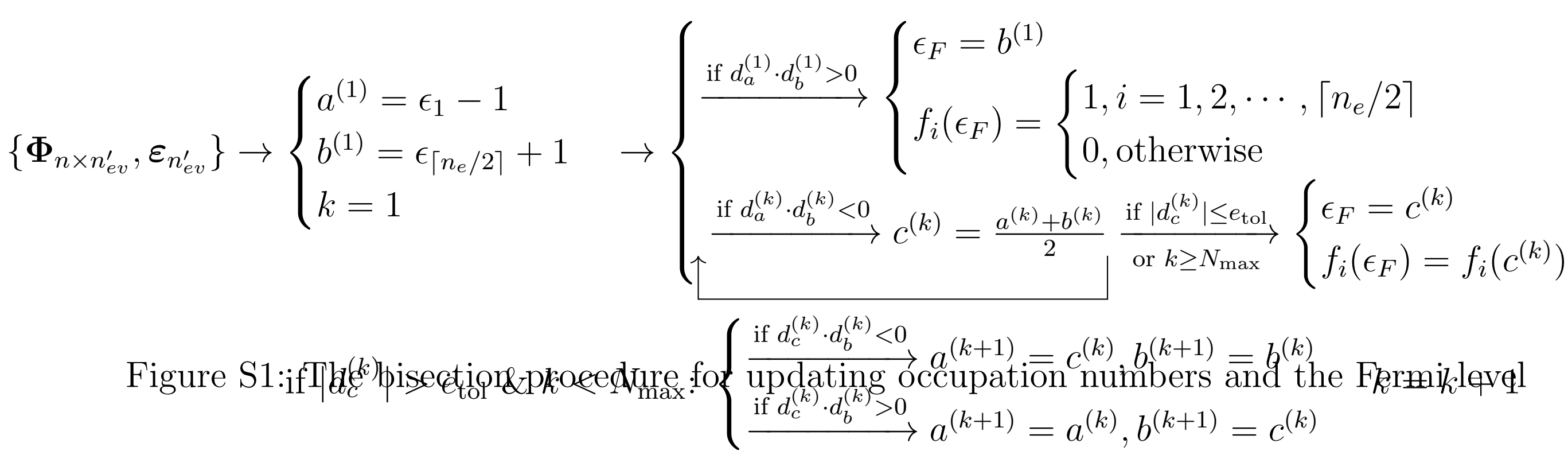

Figure S1: The bisection procedure for updating occupation numbers and the Fermi level

5 Update the Hartree potential through Poisson equation by conjugate gradient (CG) method

In continuous form, suppose the 3D simulation domain is Ω, with $S = \partial\Omega$ denotes its boundary. The homogeneous Dirichlet boundary condition indicates $u|_{\partial\Omega} = 0$ for an arbitrary sufficiently smooth nonzero real function $u \neq 0$. Integration by parts gives

$$\begin{aligned} \int_\Omega u\nabla^2 u \, d\Omega &= \int_\Omega \nabla(u\nabla u)\, d\Omega - \int_\Omega |\nabla u|^2 \, d\Omega \\ &\overset{\text{divergence}}{\underset{\text{theorem}}{=\!=\!=}} \int_{\partial\Omega} (\cancelto{0}{u}\nabla u)\cdot \vec{n}\, dS - \int_\Omega |\nabla u|^2 \, d\Omega \\ &= -\int_\Omega |\nabla u|^2 \, d\Omega < 0 \end{aligned} \tag{11}$$

where $\vec{n}$ is the outward pointing unit vector at each point on the boundary. Therefore, the Laplacian is negative definite under homogeneous Dirichlet boundary condition and the operator $-\nabla^2$ is positive definite. The FD discretization preserves this property, and therefore the matrix $\boldsymbol{L}$ is symmetric positive definite, which makes that the CG method is applicable to solve Poisson in its real space form:

$$\boldsymbol{L}\left[\Delta\vec{V}_H\right] = 4\pi\left[\Delta\vec{\rho}\right] \tag{12}$$

The CG method solves $\boldsymbol{A}\vec{x} = \vec{b}$ by minimizing the quadratic function through its gradient.

$$\phi(\vec{x}) = \frac{1}{2}\vec{x}^\mathsf{T}\boldsymbol{A}\vec{x} - \vec{b}^\mathsf{T}\vec{x} \qquad \nabla\phi(\vec{x}) = \boldsymbol{A}\vec{x} - \vec{b} \tag{13}$$

Starting from an initial guess $\vec{x}_0$, the CG algorithm proceeds as shown in Figure S2.

The iteration terminates when the relative residual $\|\mathbf{r}_k\|/\|\mathbf{r}_0\|$ falls below e_{tol} or reaches $N_{\max}$; boxed formulas are used in the numerical loop, while others are for derivation.

Compared with classical steepest (gradient) descent, the CG method converges much faster. Steepest descent moves along the negative gradient and often exhibits zigzag behavior. In contrast, CG generates mutually $\boldsymbol{A}$-conjugate directions, preserving progress from previous steps and eliminating independent spectral

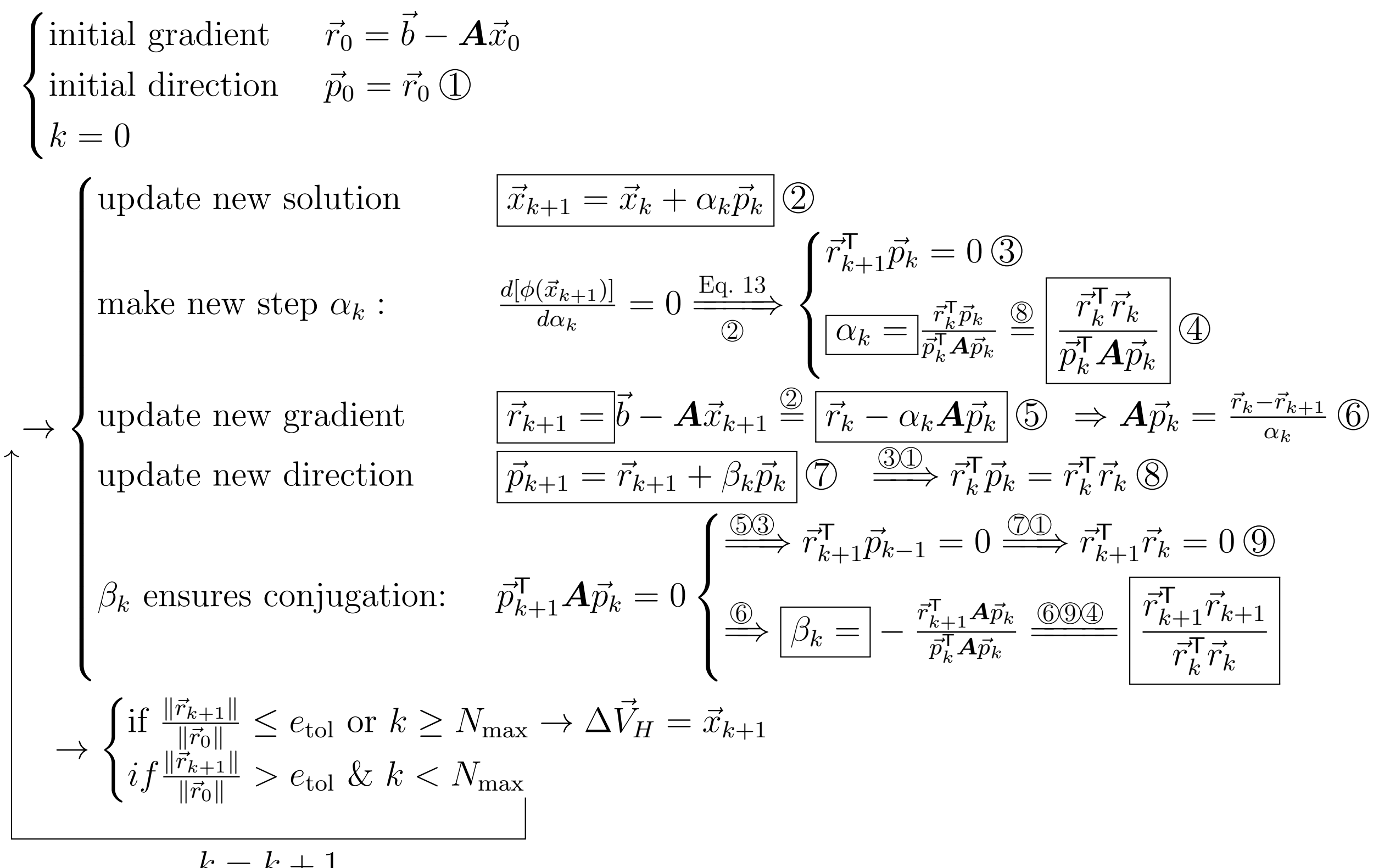

Figure S2: The CG method for updating Hartree potential through Poisson equation

error components at each iteration. This makes it especially efficient for large, sparse systems such as FD discretizations of the Poisson equation.

6 The box size convergence test for $C_{10}H_8$

To quantify the finite-domain effect, we ran a box-size convergence test on the largest molecule, $C_{10}H_8$ (molecular diameter ≈ 6.2 Å). We fixed the grid spacing at 0.2 Å and held all other parameters constant. The domain radius was increased from 4 Å to 10 Å, i.e., box lengths from 8 Å to 20 Å. Table S2 lists the resulting electronic energies per atom and HOMO–LUMO gaps. A too-small box strongly distorts both quantities. Both converge once enough vacuum is included. The main calculations leave at least ≈ 5 Å between the molecule and the boundary, which is sufficient for the conclusions here.

Table S2: Box-size convergence test for $C_{10}H_8$ with a fixed grid spacing of 0.2 Å.

Radius (Å)	E_{elec}/atom (eV)	$\lvert\Delta E_{\text{elec}}\rvert$/atom (eV)	$\lvert\Delta E_{\text{elec}}\rvert < 0.01$ eV	ϵ_{HOMO} (eV)	ϵ_{LUMO} (eV)	Gap (eV)
4	−94.54039	–	–	−5.49939	−1.94669	3.55270
5	−93.46259	1.07780	No	−5.63887	−2.15995	3.47892
6	−93.39695	0.06564	No	−5.66230	−2.19228	3.47001
7	−93.39679	0.00016	Yes	−5.67500	−2.20713	3.46787
8	−93.39973	0.00294	Yes	−5.67881	−2.21122	3.46759
9	−93.40712	0.00739	Yes	−5.68131	−2.21371	3.46760
10	−93.40164	0.00548	Yes	−5.68110	−2.21370	3.46740

7 Validation Against the Original PARSEC Code

To validate the Python implementation, we compared PARSEC.py with the original PARSEC code for the 21 molecules in the main text. We used the same geometries and the LDA functional, and matched the real-space grids, pseudopotentials, boundary conditions, and convergence settings as closely as possible. Table S3 reports the electronic energy per atom and the HOMO–LUMO gap for both codes. Across the test set, the electronic energy differs by 0.022 eV/atom on average and 0.058 eV/atom at most. The HOMO–LUMO gap differs by 0.017 eV on average and 0.045 eV at most. The two codes still differ in pseudopotential handling, grid conventions, boundary treatment, diagonalization, and SCF convergence. Agreement at this level confirms that the finite-difference Hamiltonian, nonlocal pseudopotential treatment, Poisson solver, and SCF procedure in PARSEC.py are implemented correctly.

Table S3: Validation of PARSEC.py against the original PARSEC code for the 21 molecules considered in this work.

Molecule	PARSEC energy/atom (eV)	Python energy/atom (eV)	Energy difference (eV/atom)	PARSEC HOMO–LUMO gap (eV)	Python HOMO–LUMO gap (eV)	Gap difference (eV)
$C_{10}H_8$	−93.328845	−93.376940	+0.048095	3.465011	3.466809	−0.001799
C_2H_2	−84.886894	−84.881720	−0.005174	6.883409	6.890620	−0.007211
C_2H_4	−62.113809	−62.121320	+0.007511	5.800363	5.813156	−0.012793
C_2H_6	−50.673208	−50.672240	−0.000968	7.826836	7.783351	+0.043485
C_3H_8O	−85.575447	−85.614200	+0.038753	5.679659	5.706656	−0.026997
C_6H_6	−85.545421	−85.582380	+0.036959	5.224072	5.229211	−0.005139
$CH_3CH_2CH_2OH$	−85.542852	−85.587630	+0.044778	5.745201	5.772353	−0.027152
$CH_3CH_2OCH_3$	−85.551853	−85.601160	+0.049307	5.645554	5.681551	−0.035997
CH_3CH_2OH	−93.339433	−93.348490	+0.009057	5.687837	5.702299	−0.014462
$CH_3CHOHCH_3$	−85.534988	−85.575280	+0.040292	5.424824	5.446668	−0.021844
CH_3CN	−104.633617	−104.666310	+0.032693	7.564488	7.596598	−0.032110
CH_4	−43.691942	−43.670580	−0.021362	9.233746	9.189172	+0.044575
CO	−294.565179	−294.570510	+0.005331	6.708682	6.709053	−0.000371
CO_2	−341.109017	−341.119090	+0.010073	5.262068	5.263713	−0.001645
F_2	−657.670250	−657.612130	−0.058120	5.827452	5.824870	+0.002582
H_2	−15.600392	−15.612780	+0.012388	10.635154	10.612532	+0.022622
H_2O	−154.953688	−154.982600	+0.028912	5.594732	5.585556	+0.009176
HF	−337.649985	−337.652700	+0.002715	9.008878	9.045257	−0.036379
N_2	−270.906925	−270.913350	+0.006425	8.093340	8.093136	+0.000204
NO	−351.620212	−351.623100	+0.002888	4.330849	4.333653	−0.002804
O_2	−433.327925	−433.321630	−0.006295	6.196755	6.195196	+0.001559

References

(1) Cheney, W.; Kincaid, D. Linear algebra: Theory and applications. *Aust. Math. Soc.* **2009**, *110*, 544–550.

(2) Benoit, C. Note sur une méthode de résolution des équations normales provenant de l'application de la méthode des moindres carrés à un système d'équations linéaires en nombre inférieur à celui des inconnues (Procédé du Commandant Cholesky). *Bulletin géodésique* **1924**, *2*, 67–77.

(3) Householder, A. S. Unitary triangularization of a nonsymmetric matrix. *J. ACM* **1958**, *5*, 339–342.

(4) Zhou, Y.; Saad, Y.; Tiago, M. L.; Chelikowsky, J. R. Parallel self-consistent-field calculations via Chebyshev-filtered subspace acceleration. *Phys. Rev. E* **2006**, *74*, 066704.

(5) Zhou, Y.; Saad, Y.; Tiago, M. L.; Chelikowsky, J. R. Self-consistent-field calculations using Chebyshev-filtered subspace iteration. *J. Comput. Phys.* **2006**, *219*, 172–184.

(6) Zhou, Y. A block Chebyshev–Davidson method with inner–outer restart for large eigenvalue problems. *J. Comput. Phys.* **2010**, *229*, 9188–9200.

(7) Zhou, Y.; Chelikowsky, J. R.; Saad, Y. Chebyshev-filtered subspace iteration method free of sparse diagonalization for solving the Kohn–Sham equation. *J. Comput. Phys.* **2014**, *274*, 770–782.

(8) Zhou, Y.; Li, R.-C. Bounding the spectrum of large Hermitian matrices. *Linear Algebra Appl.* **2011**, *435*, 480–493.

(9) Burden, R. L.; Faires, J. D.; Burden, A. M. *Numerical analysis*; Cengage Learning, 2016.
